# Capturing sex-specific and hypofertility-linked effects of assisted reproductive technologies on the cord blood DNA methylome

**DOI:** 10.1186/s13148-023-01497-7

**Published:** 2023-05-11

**Authors:** Sophia Rahimi, Xiaojian Shao, Donovan Chan, Josée Martel, Anick Bérard, William D. Fraser, Marie-Michelle Simon, Tony Kwan, Guillaume Bourque, Jacquetta Trasler

**Affiliations:** 1 Research Institute of the McGill University Health Centre, Montreal, QC Canada; 2 Digital Technologies Research Centre, National Research Council Canada, Ottawa, ON Canada; 3 Department of Biochemistry, Microbiology and Immunology, University of Ottawa, Ottawa, ON Canada; 4 Research Unit On Medications and Pregnancy, Research Centre, CHU Sainte-Justine, Montreal, Canada; 5 Faculty of Pharmacy, Université de Montréal, Montreal, QC Canada; 6 Faculty of Medicine, Université Claude Bernard Lyon 1, Lyon, France; 7 Department of Obstetrics and Gynecology, Université de Sherbrooke and Centre de Recherche du CHUS, Sherbrooke, QC Canada; 8 McGill University Genome Centre, Montreal, QC Canada; 9 Department of Human Genetics, McGill University, Montreal, QC Canada; 10 Department of Pharmacology and Therapeutics, McGill University, Montreal, QC Canada; 11 Department of Pediatrics, McGill University, Montreal, QC Canada

**Keywords:** Assisted reproductive technology, Genomic imprinting, Sex specific, Infertility, Hypofertility, DNA methylation, Cord blood

## Abstract

**Background:**

Children conceived through assisted reproduction are at an increased risk for growth and genomic imprinting disorders, often linked to DNA methylation defects. It has been suggested that assisted reproductive technology (ART) and underlying parental infertility can induce epigenetic instability, specifically interfering with DNA methylation reprogramming events during germ cell and preimplantation development. To date, human studies exploring the association between ART and DNA methylation defects have reported inconsistent or inconclusive results, likely due to population heterogeneity and the use of technologies with limited coverage of the epigenome. In our study, we explored the epigenetic risk of ART by comprehensively profiling the DNA methylome of 73 human cord blood samples of singleton pregnancies (*n* = 36 control group, *n* = 37 ART/hypofertile group) from a human prospective longitudinal birth cohort, the 3D (Design, Develop, Discover) Study, using a high-resolution sequencing-based custom capture panel that examines over 2.4 million autosomal CpGs in the genome.

**Results:**

We identified evidence of sex-specific effects of ART/hypofertility on cord blood DNA methylation patterns. Our genome-wide analyses identified ~ 46% more CpGs affected by ART/hypofertility in female than in male infant cord blood. We performed a detailed analysis of three imprinted genes which have been associated with altered DNA methylation following ART (*KCNQ1OT1*, *H19/IGF2* and *GNAS*) and found that female infant cord blood was associated with DNA hypomethylation. When compared to less invasive procedures such as intrauterine insemination, more invasive ARTs (in vitro fertilization, intracytoplasmic sperm injection, embryo culture) resulted in more marked and distinct effects on the cord blood DNA methylome. In the in vitro group, we found a close to fourfold higher proportion of significantly enriched Gene Ontology terms involved in development than in the in vivo group.

**Conclusions:**

Our study highlights the ability of a sensitive, targeted, sequencing-based approach to uncover DNA methylation perturbations in cord blood associated with hypofertility and ART and influenced by offspring sex and ART technique invasiveness.

**Supplementary Information:**

The online version contains supplementary material available at 10.1186/s13148-023-01497-7.

## Background

Assisted reproductive technologies (ARTs) are widely used to help couples overcome infertility and other barriers to build their families. In fact, up to 7.9% of live births in developed countries can be attributed to the use of ART [1]. Although the majority of children conceived using these techniques are born healthy, studies have shown that this population is at an increased risk for adverse health outcomes, including rare genomic imprinting disorders [2]. Importantly, the timing of ART coincides with critical DNA methylation reprogramming events during germ cell and preimplantation development. Therefore, the increased risk for health issues among the ART-conceived population may be related to epigenetic instability [3].

Despite concerns regarding the epigenetic impact of ART on infants for several years, the association between ART and DNA methylation defects remains unclear due to contradictory reports in the literature. Recently, Barberet et al. [4] conducted a systematic review and meta-analysis according to source tissue and time of sample collection. Although the authors acknowledge that several individual studies do not report any ART-induced epigenetic changes, their meta-analysis concluded that DNA methylation abnormalities are associated with ART in a tissue-specific manner, particularly for imprinted genes [4]. Nonetheless, the authors note that the sites altered by ART in epigenome-wide studies are poorly replicated using targeted techniques, which is likely due to heterogeneity in study populations and methodologies. Therefore, further research utilizing standardized methods is needed to fully characterize the epigenetic influence of assisted reproduction.

There are several different ART treatments that vary in the nature of their manipulations, from more simple treatments such as ovarian stimulation to more invasive intracytoplasmic sperm injection (ICSI). Mouse [5, 6] and human [7, 8] studies indicate that distinct ART types have unique epigenetic effects, specifically with more invasive procedures (often involving in vitro manipulations and culture) being associated with greater epigenetic harm. Hence, the effect of ART may be difficult to ascertain. Moreover, there is the added effect of parental infertility, which may confound the observed effects of ART. It has been reported that epigenetic defects in the sperm of infertile/subfertile men can be transmitted to offspring and have been associated with adverse (or abnormal) pregnancy outcomes [9]. In addition, a recent study examining cord blood from twin pregnancies identified DNA methylation changes likely induced by parental subfertility rather than in vitro fertilization (IVF) [10]. This is further supported by the recent finding that the epigenetic dysregulation associated with infertility may differ based on the time to pregnancy [11]. Therefore, an ongoing challenge in human studies is differentiating the epigenetic consequence of different ART types, as well as the contribution of parental infertility.

Mouse [6] and human [12] studies have supported the hypothesis that only a subset of conceptuses is susceptible to ART-induced epigenetic defects. This implies that the majority of ART-conceived offspring will present as epigenetically similar to those who are conceived naturally, rendering it difficult to capture the defects for which certain ART infants are at risk. Our group recently provided further evidence in support of this hypothesis; although we did not identify specific CpGs that are differentially methylated between placentas from ART and control pregnancies, a group of outlier samples enriched in the former was identified [8]. Therefore, it is plausible that the contradictory reports in human ART studies may be attributed to the existence of such “epigenetic outliers”.

The aim of this study was to determine the effect of ART/hypofertility on DNA methylation patterning in cord blood from singleton pregnancies using the Quebec-based Canadian 3D (Design, Develop, Discover) longitudinal pregnancy cohort [13]. Genome-wide DNA methylation analysis was performed using a 5-methylcytosine capture sequencing (MCC-seq) panel covering over 3 million CpGs, including sites proven to be environmentally sensitive [14], with the goal of fully characterizing the DNA methylation disturbances induced by ART/hypofertility at birth. We chose to examine cord blood, a proxy for foetal tissues, and hypothesized that it will provide insight into the mechanism by which infants may be impacted by assisted conception.

## Results

### Cohort information

In this study, 73 cord blood samples from the Quebec-based Canadian 3D longitudinal pregnancy cohort [13] were classified into two groups based on the fertility status/conception method. The control group consisted of samples collected from pregnancies without a parental history of subfertility, in which natural conception was achieved in less than 6 months (*n* = 36); samples from pregnancies with clinically diagnosed parental infertility and/or conception achieved using ART were classified into the ART/hypofertile group (*n* = 37). The cohort demographics and birth characteristics for both populations are summarized in Table 1. No significant differences were observed between the ART/hypofertile and control groups in terms of parental [maternal age at delivery, paternal age, maternal body mass index (BMI)] or birth (gestational age, infant sex ratio) characteristics. Prematurity was defined as a gestational age < 37 weeks, and in our study the gestational age of samples categorized as premature was 35–36 weeks. The ART/hypofertile group consisted of samples from two subpopulations defined by the conception method’s level of invasiveness: samples collected from pregnancies conceived in vivo (in vivo subgroup, *n* = 17) and in vitro (in vitro subgroup, *n* = 20). The in vivo group comprised cord blood samples from couples with clinically defined infertility who conceived spontaneously after more than 1 year of unprotected intercourse (> 1 yr, *n* = 8) or couples who conceived through intrauterine insemination (IUI, *n* = 9). In contrast, the in vitro group included samples from pregnancies achieved using more invasive techniques: in vitro fertilization (IVF, *n* = 4) or intracytoplasmic sperm injection (ICSI, *n* = 16), both of which are followed by embryo culture and transfer (refer to Table 2 for details about the in vivo and in vitro groups).

**Table 1:** Description of the control and ART/hypofertile groups

	Control group n = 36	ART/hypofertile group n = 37	*p* Value
Maternal age in years	34.1 (3.3)	33.8 (4.5)	0.70
Paternal age in years	36.7 (5.3)	37.7 (7.1)^a^	0.48
Maternal BMI	24.5 (5.0)^b^	24.8 (5.1)^c^	0.81
Smoking status during pregnancy (S/SSP/NS^d^)	3/7/26	3/5/29	0.79
*Obstetrical issues (M/F*^*e*^*)*			
Normal	27 (14/13)	25 (14/11)	0.30
Prematurity alone	5 (1/4)	2 (0/2)	
Gestational diabetes (GD) alone	2 (1/1)	4 (4/0)	
Pre-eclampsia alone	2 (1/1)	2 (0/2)	
Pre-eclampsia + prematurity	0	3 (1/2)	
Pre-eclampsia + GD	0	1 (0/1)	
Delivery route (Vaginal/C section)	26/10	23/14	0.46
Gestational age in weeks^f^	38.7 (1.6)	38.6 (1.6)	0.79
Infant sex (M/F^e^)	17/19	19/18	0.82
*ART/hypofertile subgroup*^*g*^			
In vivo (> 1 yr/IUI)	–	17 (8/9)	–
In vitro (IVF/ICSI)	–	20 (4/16)	–

^a^1 Paternal age unknown

^b^1 BMI unknown

^c^5 BMI unknown

^d^S, smoking during pregnancy; SSP, stopped smoking before pregnancy; NS, no smoking

^e^M, male; F, female

^f^10 were considered to be premature (< 37 weeks gestational age at delivery): 7/10 were delivered at 36 weeks (4 control and 3 ART/hypofertile) and 3/10 were delivered at 35 weeks (1 control and 2 ART/hypofertile)

^g^ > 1 yr, spontaneous pregnancy after > 1 year of unprotected intercourse; IUI, intrauterine insemination; IVF, in vitro fertilization; ICSI, intracytoplasmic sperm injection

For numerical variables, mean (SD) is shown and two-tailed unpaired *t* test was performed to compare groups

For categorical variables, *N* is shown and Fisher’s exact test or chi-square test was performed to compare groups

**Table 2:** Description of the ART/hypofertile in vivo and in vitro subgroups

	In vivo group n = 17	In vitro group n = 20	*p* Value
ART technique (ICSI/IVF^a^)	–	16/4	–
Number with blastocyst transfers	–	20	–
*Aetiology for hypofertility*			
Male	2	9	0.27
Female	3	3	
Male + female	2	1	
Unexplained	2	7	
*Obstetrical issues (M/F*^*b*^*)*			
Normal	13 (8/5)	12 (6/6)	0.58
Prematurity alone	1 (0/1)	1 (0/1)	
Gestational diabetes (GD) alone	2 (2/0)	2 (2/0)	
Pre-eclampsia alone	1 (0/1)	1 (0/1)	
Pre-eclampsia + prematurity	0	3 (1/2)	
Pre-eclampsia + GD	0	1 (0/1)	
Spontaneous conception after > 1 year of unprotected intercourse	8	–	–
Infant sex (M/F^b^)	10/7	9/11	0.51

^a^ICSI, intracytoplasmic sperm injection; IVF, in vitro fertilization

^b^M, male; F, female

For all variables, N is shown and fisher’s exact test or chi-square test was performed to compare groups

### MCC-seq reveals sex-specific genome-wide DNA methylation perturbations associated with ART/hypofertility

To assess the effect of ART/hypofertility on genome-wide DNA methylation in cord blood, we profiled the DNA methylome using MCC-seq, a capture panel that provides sequencing-based information on ~ 3.2 million CpGs, including the > 850,000 sites present on the Infinium MethylationEPIC BeadChip as well as additional sites (~ 1 million) proven to be environmentally sensitive [14]. Specifically, 3,151,021 CpGs were measured in at least one sample at the 1 × read coverage. A total of 2,991,643 CpGs were obtained after removing SNPs and blacklisted overlapping CpGs. When limited to CpGs with read coverage between 15× (inclusive) and 500× (inclusive), 2,905,698 CpGs covered in at least one sample were generated, which were further reduced to 2,501,977 CpGs after requiring coverage for ≥ 30 samples. After removing CpGs located on sex chromosomes, 2,464,592 were retained for downstream analysis. A generalized linear model was applied for these remaining CpGs to identify differentially methylated CpGs (DMCs) between ART/hypofertile and control samples.

**Fig. 1:**
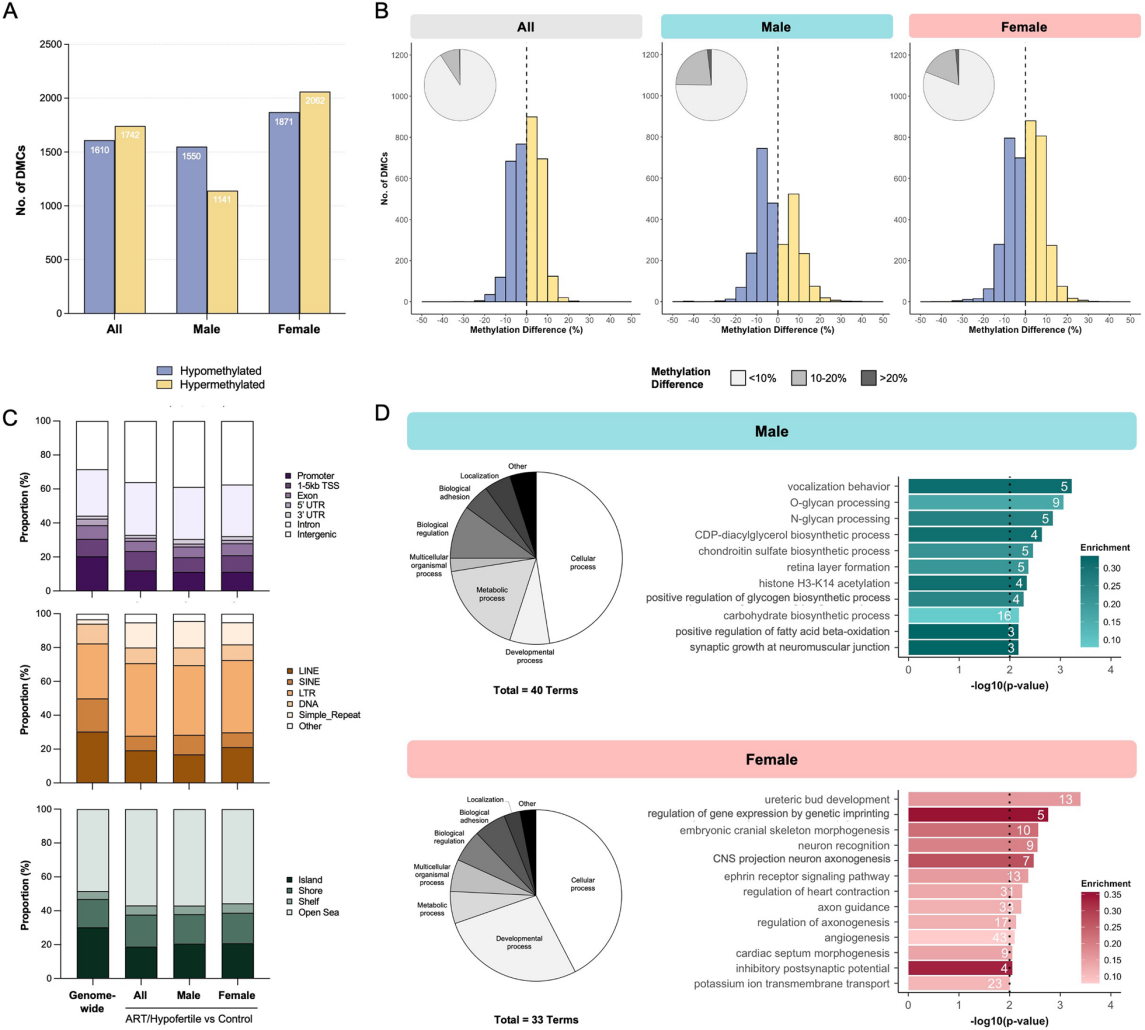
ART/hypofertility differentially affects genome-wide DNA methylation in the cord blood of male and female infants. **A** Total number of hypomethylated and hypermethylated DMCs between ART and control groups when all, only male and only female samples were used for comparison. **B** Methylation differences at DMCs between ART and control groups, with pie charts summarizing the proportion of DMCs demonstrating less than 10%, between 10 and 20% and greater than 20% methylation differences. **C** Distribution of DMCs within genomic regions (top), repetitive elements (middle) and CpG-rich regions (bottom) in comparison to all captured sites genome-wide. **D** Gene Ontology analysis of all genic DMCs was performed. Pie charts demonstrating the categorical distribution of all significant biological processes for the ART versus Control analysis in males and females and selected terms are shown. The number of observed genes associated with each term is indicated within bars. Enrichment is defined as the proportion of observed genes compared to the number annotated within the whole dataset and is indicated by bar colour. The dotted line represents the significance threshold: weighted Fisher’s *p* value < 0.01

Our initial comparison (herein referred to as “combined analysis”) included all 73 cord blood samples and identified a total of 3352 DMCs significantly altered due to ART/hypofertility (1610 hypomethylated and 1742 hypermethylated DMCs) (Fig. 1A). We also performed sex-stratified analyses by examining the cord blood of male (*n*_control_ = 17, *n*_ART/hypofertile_ = 19) and female (*n*_control_ = 19, *n*_ART/hypofertile_ = 18) newborns separately to determine whether the effect of ART/hypofertility differs based on infant sex. Notably, ART/hypofertility was associated with alterations at fewer DMCs in male infant cord blood (2691 total; 57.6% hypo- and 42.4% hypermethylated) than in female infant cord blood (3933 total; 47.6% hypo- and 52.4% hypermethylated; Fig. 1A). Although the vast majority of DMCs (for all 3 analyses) exhibited subtle differences between the groups (Fig. 1B), a higher proportion of DMCs identified by sex-stratified analyses showed methylation differences of > 10% (24.8% and 19.1% of DMCs in males and females, respectively) compared to those identified using combined analysis (9.2% of DMCs) (Fig. 1B). In comparison to the ~ 2.4 million capture panel sites remaining after sequencing data processing (genome-wide background; refer to Methods section for details), DMCs affected by ART/hypofertility (for all 3 analyses) were enriched for intergenic regions and introns (Fig. 1C, top), long terminal repeats (LTRs) and simple repeats (Fig. 1C, middle) but depleted for CpG islands (Fig. 1C, bottom).

We were interested in whether pregnancy complications (prematurity, gestational diabetes and pre-eclampsia), which are found in both our control and ART/hypofertility groups (though not significantly different, Table 1), may influence our results. We therefore carried out an additional analysis, adding as a covariate the presence or absence of any pregnancy complications. The results demonstrated that a large majority of DMCs in this new analysis (3146 of 3341 DMCs discovered) were in common with those in the original analysis (3352 DMCs); these were all found to be affected in the same direction (data not shown). A sex-stratified analysis including pregnancy complications as a covariate was also performed to compare the control and ART/hypofertile groups in males and females separately. For the males, we discovered 2679 DMCs, a similar number to that in the original analysis (2691 DMCs) with a similar proportion of DMCs that were hyper/hypo; approximately 87% of the DMCs were in common and showing the same directionality (i.e. both demonstrated hyper- or hypomethylation) (data not shown). For the female-only results, our re-analysed covariate analysis demonstrated 3921 DMCs (compared to 3933 DMCs in the original female results); again, the proportion of DMCs that was hyper/hypo were similar between the two analyses. Similarly, 3505 DMCs (89% overlap) were found in common between these two analyses, with all sites of differential methylation showing alterations in DNA methylation in the same direction (data not shown).

Gene Ontology enrichment analysis was performed using male and female genic DMCs. At a significance threshold of *p* < 0.01, 40 and 33 biological processes were significantly enriched among the male and female genic DMCs, respectively. Although cellular processes represented the highest proportion of enriched pathways in both sexes, we observed that the next most abundant type was metabolic processes for males and developmental processes for females (Fig. 1D). More specifically, the genic DMCs affected by ART/hypofertility in male infant cord blood showed enrichment for carbohydrate and carbohydrate-derivative metabolic processes (Fig. 1D). In contrast, the developmental processes enriched among genic DMCs in females are predominantly related to nervous system and circulatory system development (Fig. 1D). Gene Ontology enrichment and gene network analyses were also performed by separately assessing hypo- and hypermethylated genic DMCs in males and females (refer to Additional file 1: Fig. S1A, B for details). Notably, hypomethylated DMCs in females were significantly enriched for pathways related to epigenetic regulation (Additional file 1: Fig. S1A).

**Fig. 2:**
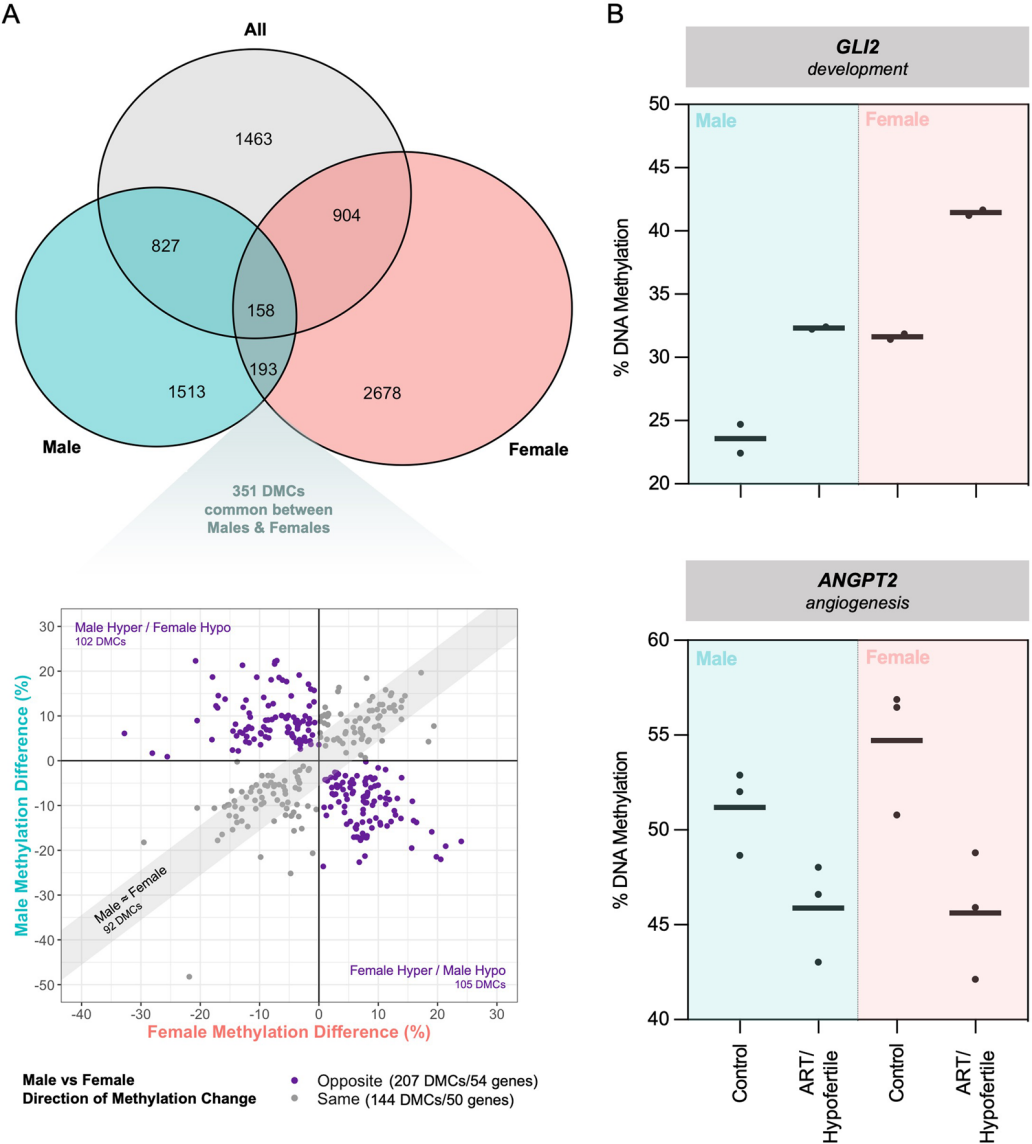
Common effects of ART/hypofertility in males and females. **A** Venn diagram showing sites commonly affected by ART using all, only male and only female samples. Methylation differences between the ART and control groups for DMCs common between males and females are shown. DMCs demonstrating methylation changes in the same or opposite direction for both sexes are indicated in grey and purple, respectively. DNA methylation values at 92 DMCs are similar between male and female infant cord blood. Methylation difference values were deemed to be similar if they were within ± 5% (this range is shaded in grey). **B** ART-induced methylation changes at selected genes commonly affected by ART in males and females. Each dot represents a CpG that is significantly differentially methylated in both sexes, with the mean value for all samples within the group indicated. The thick horizontal line represents the mean of all CpGs shown

By comparing the list of DMCs, only 36.6% (985) and 27.0% (1062) of male and female DMCs, respectively, were identified in combined analysis (Fig. 2A). In addition, a relatively small number of sites (351 or 5.3%) were altered by ART/hypofertility in both males and females. When the ART/hypofertility-induced methylation differences in males and females were plotted, we observed the opposite direction of change between the sexes for 59.0% of these common sites (Fig. 2A). Taken together, these data provide insight into the nature of sex-specific ART/hypofertility effects, revealing that not only are different sites vulnerable to ART/hypofertility in males and females but that even shared sites differ between sexes in terms of the direction of induced methylation changes.

Although small in number, we examined common sites between males and females with the same direction of induced methylation changes. We found that these DMCs are annotated to important genes such as *GLI2* and *ANGPT2*, which are associated with development and angiogenesis, respectively (Fig. 2B). Therefore, despite the sex-specific differences described above, there are likely important common effects of ART/hypofertility in males and females.

### Merging sites into regions reveals biologically important areas of the genome affected by ART/hypofertility

**Fig. 3:**
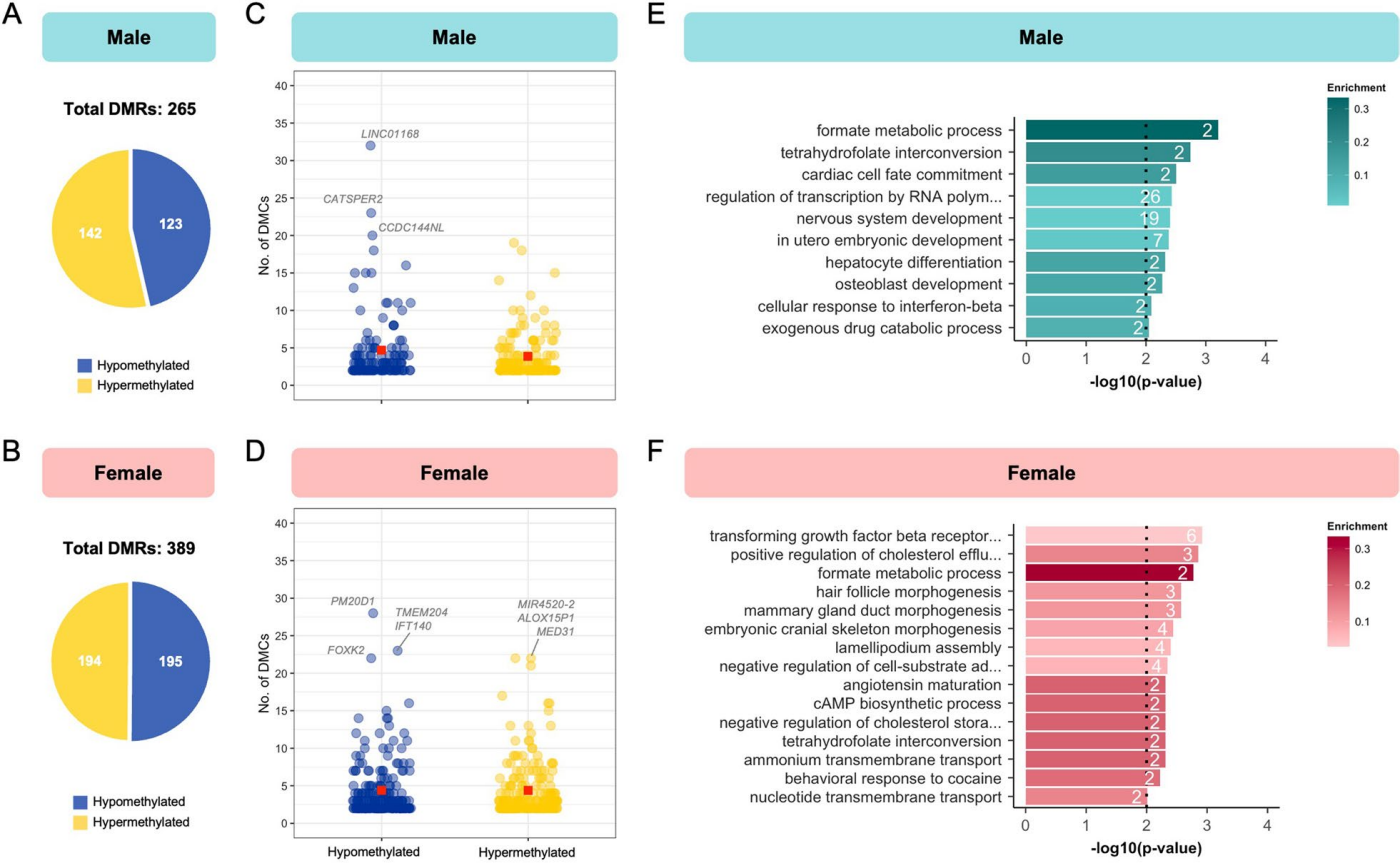
ART/hypofertility affects DNA methylation in regions of biological importance. Number of hypo- and hypermethylated differentially methylated regions (DMRs) between ART and control groups for **A** males and **B** females. Number of DMCs merged within each hypo- and hypermethylated DMR for **C** males and **D** females. The average number of DMCs/region is indicated by a red square. Genes annotated to DMRs containing the highest number of DMCs are identified. All significant biological processes from Gene Ontology analysis on all DMRs located within genic regions for the ART versus Control analysis in **E** males and **F** females. The number of observed genes associated with each term is indicated within bars. Enrichment is defined as the proportion of observed genes compared to the number annotated within the whole dataset and is indicated by bar colour. The dotted line represents the significance threshold: weighted Fisher’s *p* value < 0.01

Next, we merged neighbouring sites exhibiting differential methylation (within 250 bp) into regions because they are more likely to have functional consequences than isolated DMCs. Notably, hypomethylated and hypermethylated DMCs were merged separately into differentially methylated regions (DMRs). Upon merging the sites altered by ART/hypofertility in males and females into regions, 58% (973 hypo- and 592 hypermethylated sites; Additional file 1: Fig. S2A, B left) and 57% (1013 hypo- and 1212 hypermethylated sites; Additional file 1: Fig. S2C, D left) of DMCs remained as single CpG sites, respectively. Overall, 265 (123 hypo- and 142 hypermethylated; Fig. 3A) and 389 (195 hypo- and 194 hypermethylated; Fig. 3B) DMRs were identified in males and females, respectively. The average sizes of hypo- and hypermethylated regions affected by ART/hypofertility in females (118 bp and 126 bp, respectively; Additional file 1: Fig. S2C, D right) were slightly larger than those in males (110 bp and 95 bp, respectively; Additional file 1: Fig. S2A, B right).

By plotting the number of DMCs per hypo- and hypermethylated DMR, we were able to assess which regions are most affected by ART/hypofertility (Fig. 3C, D). The regions most impacted by ART/hypofertility in males are annotated to the following genes: *LINC01168*, *CATSPER2* and *CCDC144NL* (Fig. 3C). For females, highly affected DMRs are annotated to *PM20D1*, *TMEM204, IFT140, FOXK2, MIR4520-2*, *ALOX15P1*, and *MED31* (Fig. 3D). Gene Ontology enrichment analysis of genes annotated to male and female DMRs revealed 10 and 15 significant biological processes, respectively (Fig. 3E, F). Developmental processes, including nervous system development and in utero embryonic development, were among the pathways enriched in the regions affected by ART/hypofertility in males (Fig. 3E). Regions associated with ART/hypofertility in females were enriched for various biological processes, including those involved in anatomical structure development (Fig. 3F). Interestingly, comparing the results of GO enrichment analysis for DMCs and DMRs revealed little to no overlap between the terms within each infant sex group. Overall, these results suggest that some regions in the genome are particularly vulnerable to ART/hypofertility-induced epigenetic aberrations and that these regional changes may be associated with different biological consequences in males and females.

### DNA methylation at imprinting control regions is affected by ART/hypofertility in a sex-specific manner

Imprinted loci have been reported to be particularly vulnerable to ART-induced epigenetic perturbations [4]. The MCC-seq panel allowed for examination of 50 ICRs (refer to Additional file 2, tab 1 for information about the number of CpGs covered by the MCC-seq panel for each ICR). Very few sites within imprinting control regions (ICRs) were identified as significantly affected by ART/hypofertility in our differential methylation analysis; of the 50 ICRs that were examined, only 2 showed CpGs (5 total) affected by ART/hypofertility (in the combined analysis). In the combined analysis, *VTRNA2-1* (3 DMCs) showed ~ 7.0% hypomethylation in the ART/hypofertile group compared to Control, while *WDR27* (2 CpGs) demonstrated ~ 3.5% hypomethylation (Additional file 2, tab 2). To determine if ICR hypomethylation was a common trend associated with ART/hypofertility, we investigated average DNA methylation across all ICRs (Additional file 2,  tab 3). In our combined analysis, 32 of 49 ICRs with data showed hypomethylation associated with ART/hypofertility. There was also evidence of sex-specific effects, with 35 out of 49 ICRs more hypomethylated in females than in males; in addition, for 25 ICRs, hypomethylation was found in females and hypermethylation in males.

Having demonstrated trends for hypomethylation in ICRs of imprinted genes, and sex-specific effects, we were interested in examining, in greater detail, the ICRs for two imprinted genes for which disruptions in DNA methylation account for the majority of Beckwith-Wiedemann syndrome cases in the ART-conceived population: *KCNQ1OT1* and *H19/IGF2* ICRs [15]. In addition, we assessed four ICRs for different *GNAS* transcripts, as according to 3D cohort data, this imprinted locus is highly affected by ART/infertility in the placenta [8]. We therefore, examined all individual sites for the 6 ICRs of these 3 imprinted genes to investigate whether average DNA methylation across specific ICRs is significantly different between ART/hypofertile and control groups. Information about these ICRs is summarized in Additional file 1: Table S1, including their genomic coordinates, parental origin, type (i.e. whether they are oocyte gametic, sperm gametic or secondary DMRs) and the total number of CpGs present within these regions. The use of MCC-seq allows for this type of analysis to be informative because it provides data on a higher percentage of total CpGs for ICRs (32.5–82.8%) compared to the commonly used Illumina 450 K array (3.0–48.4%) (Additional file 1: Table S1).

**Fig. 4:**
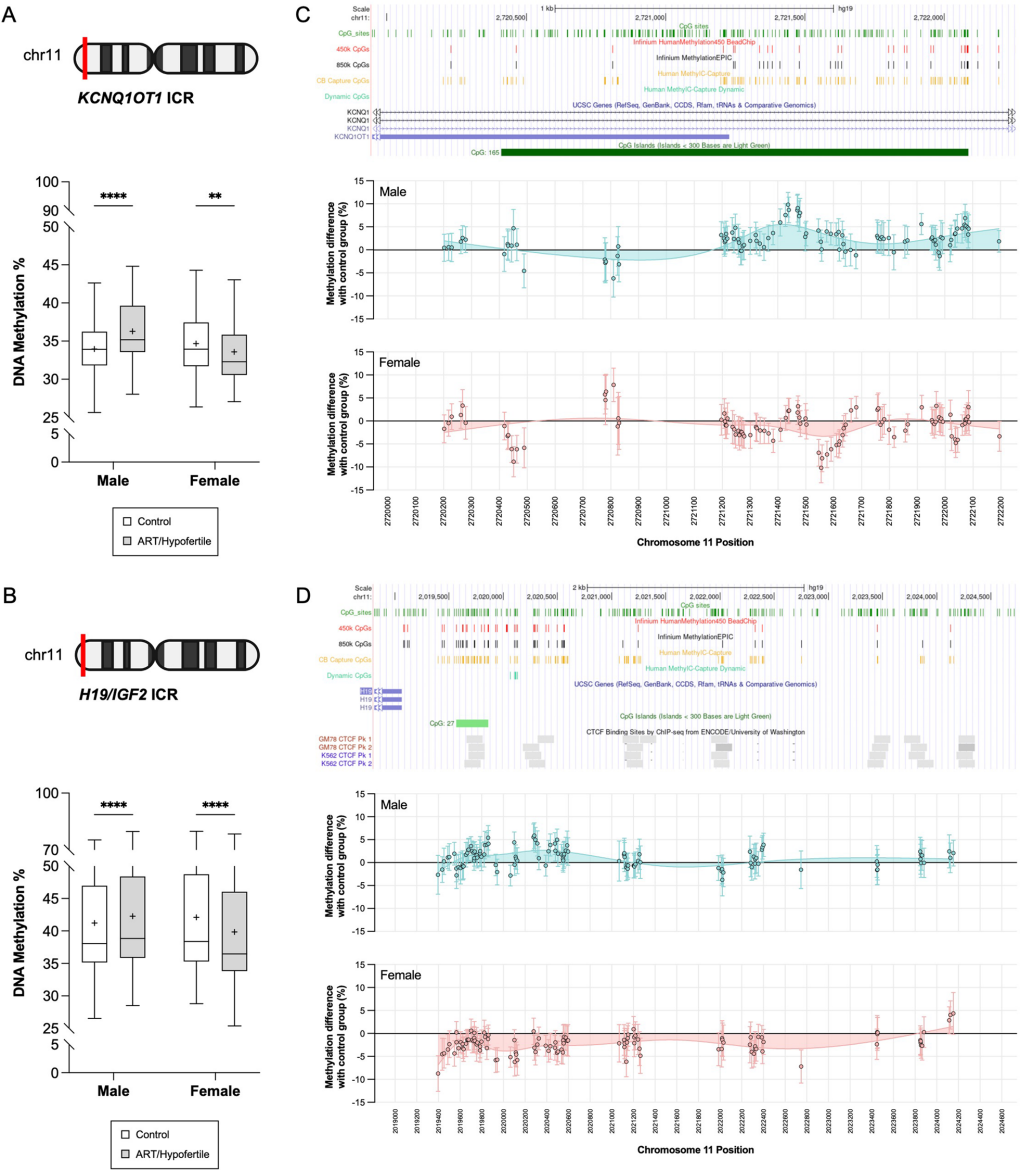
Sex-specific effect of ART/hypofertility on DNA methylation at imprinting control regions (ICRs). DNA methylation within the maternally methylated *KCNQ1OT1* ICR (97 CpGs) (**A**) and paternally methylated *H19/IGF2* ICR (106 CpGs) (**B**) was compared between ART and control groups after sex stratification. To avoid potential skewing caused by missing data, methylation at each CpG was averaged across samples for each CpG. Box plot bodies extend from the 25th to 75th percentiles, with the whiskers extending to the minimum and maximum data values; + represents the mean, and the line within the box represents the median of all CpGs. Two-way ANOVA with Bonferroni correction for multiple comparisons was used to compare ART and control groups for males and females; **p < 0.01, ****p < 0.0001. UCSC Genome Browser view of the ICR of *KCNQ1OT1* (**C**) and *H19/IGF2* (**D**) with DNA methylation differences between ART and control groups shown for each captured CpG. Custom tracks indicate CpG sites (green), CpGs analysed by the Illumina HumanMethylation450 array (red), CpGs analysed by the Illumina HumanMethylationEPIC array (black), the sites captured in our study by the MCC-seq capture (gold), and Sperm Dynamic CpG sites (light green). A data point represents the methylation difference between ART and control groups at a CpG, which was calculated by averaging all replicates for each group and using the mean values to compute the difference (ART-Control). Means ± SEMs values are shown, and each graph contains a smoothing spline curve (using 8 knots) to demonstrate the overall methylation difference trend across the loci

When comparing the ART/hypofertile and control groups, we observed subtle yet significant sex-specific differences in average DNA methylation at *KCNQ1OT1* and *H19/IGF2* DMRs after sex stratification. More specifically, at both imprinted loci, ART/hypofertility was associated with hypermethylation in males and hypomethylation in females (Fig. 4A, B). To determine whether this effect is driven by a subset of CpGs, we examined the methylation of all highly covered CpGs within those regions and found that sex-specific methylation changes induced by ART/hypofertility occurred at the majority of CpGs (Fig. 4C, D). Sex-specific effects were also observed for the *GNAS* DMRs, being more marked in females. Compared to control samples, ART/hypofertile samples were significantly hypermethylated at the *GNAS-NESP* DMR (Additional file 1: Fig. S3A) and hypomethylated at the *GNAS-XL* DMR in males (Additional file 1: Fig. S3C)*.* In contrast, ART/hypofertility was associated with altered DNA methylation (specifically hypomethylation) at *GNAS-NESP*, *GNAS-XL* and *GNAS-A/B* DMRs in females (Additional file 1: Fig. S3A, C, D). Notably, methylation at the *GNAS-AS1* DMR was unaffected by ART/hypofertility for both sexes (Additional file 1: Fig. S3B). Once again, we wanted to determine whether pregnancy complications (prematurity, gestational diabetes and pre-eclampsia) may be influencing our results. Therefore, we re-examined the *KCNQ1OT1* ICR having excluded participants with pregnancy complications. As was observed in our original analysis, ART/hypofertility was associated with hypermethylation in males and hypomethylation in females (data not shown). In summary, ART/hypofertility differentially affects males and females at the *KCNQ1OT1* ICR, *H19/IGF2* ICR, *GNAS-NESP* DMR and *GNAS-A/B* DMR.

### Effect of ART/hypofertility is not driven by enrichment of outlier DNA methylation within any experimental or sex group

Our previous study examining the effect of ART/infertility on the placental DNA methylome using the 3D cohort revealed that a subset of offspring (herein referred to as “outliers”) are susceptible to DNA methylation abnormalities and that they are predominantly female offspring or from the ART/infertile group [8]. To determine whether enrichment of outliers in one group over the other is responsible for the observed differential methylation in cord blood, we employed the same outlier detection procedure (refer to “Methods” section for details). Briefly, this involved performing a series of principal component analyses (PCAs) and using the “at most one change” (AMOC) method to identify outliers based on gap sizes between samples along principal component 1 (PC1). It is important to note that we utilized only sites covered by all 73 cord blood samples for all generated PCAs.

PCA was performed using all autosomal sites (799,724 CpGs, Additional file 1: Fig. S4A) as well as only autosomal sites present on the Illumina 450 K array (95,635 CpGs, Additional file 1: Fig. S4D). The same two outlier samples were detected along PC1 for both analyses (Additional file 1: Fig. S4A, B, D, E), with neither the experimental group nor the sex group being enriched for outlier samples (Additional file 1: Fig. S4C, F).

Choufani et al. [8] also reported placentas corresponding to female offspring and from the ART/infertile group to be enriched for abnormal DNA methylation at imprinted loci and that PCA using only probes overlapping ICRs is able to identify the majority of the same outliers. To further investigate whether cord blood in the ART/hypofertile group or corresponding female offspring are more susceptible to aberrant DNA methylation, we performed PCA and outlier detection using all capture sites overlapping ICRs (1087 CpGs, (Additional file 1: Fig. S5A, B) and only sites overlapping ICRs that are present on the Illumina 450 K array (290 CpGs, Additional file 1: Fig. S5D, E). Neither analysis identified significant outlier enrichment within the ART/hypofertile group or among cord blood corresponding to female offspring (Additional file 1: Fig. S5C, F). Overall, the epigenetic outlier phenomenon identified in the placenta was not detected using cord blood DNA methylation.

### Differential effect of in vivo and in vitro ARTs on genome-wide DNA methylation

Similar to Choufani et al. [8], we sought to explore whether methylation changes associated with ART/hypofertility are dependent on the technique used to achieve conception. To perform this analysis, we separated the ART/hypofertile group into two subgroups based on whether the samples were collected from pregnancies conceived in vivo (*n* = 17) or in vitro (*n* = 20). We compared the DNA methylation profiles between the subgroups and found 3406 sites with differential methylation, with the majority showing increased methylation in the in vitro group compared to the in vivo group (1441 hypo- vs. 1965 hypermethylated DMCs; Additional file 1: Fig. S6A).

In the placentas from this cohort, a subset of the in vitro group enriched for male factor aetiology infertility and advanced paternal age clustered away from other samples within this group, suggesting interaction between infertility and ART techniques in affecting the placental methylome [8]. To determine whether such an interaction is also present for the cord blood DNA methylome, PCA utilizing only the 2016 DMCs with complete data (Additional file 1: Fig. S6B) and unsupervised hierarchical clustering was performed (Additional file 1: Fig. S6C). Both analyses highlighted the difference between the in vivo and in vitro groups at the identified DMCs, as both groups clustered away from one another (Additional file 1: Fig. S6B, C). Contrary to the placenta, no subset of cord blood samples enriched in any particular clinical characteristic clustered away from the other samples within either the in vivo or in vitro group (Additional file 1: Fig. S6C).

**Fig. 5:**
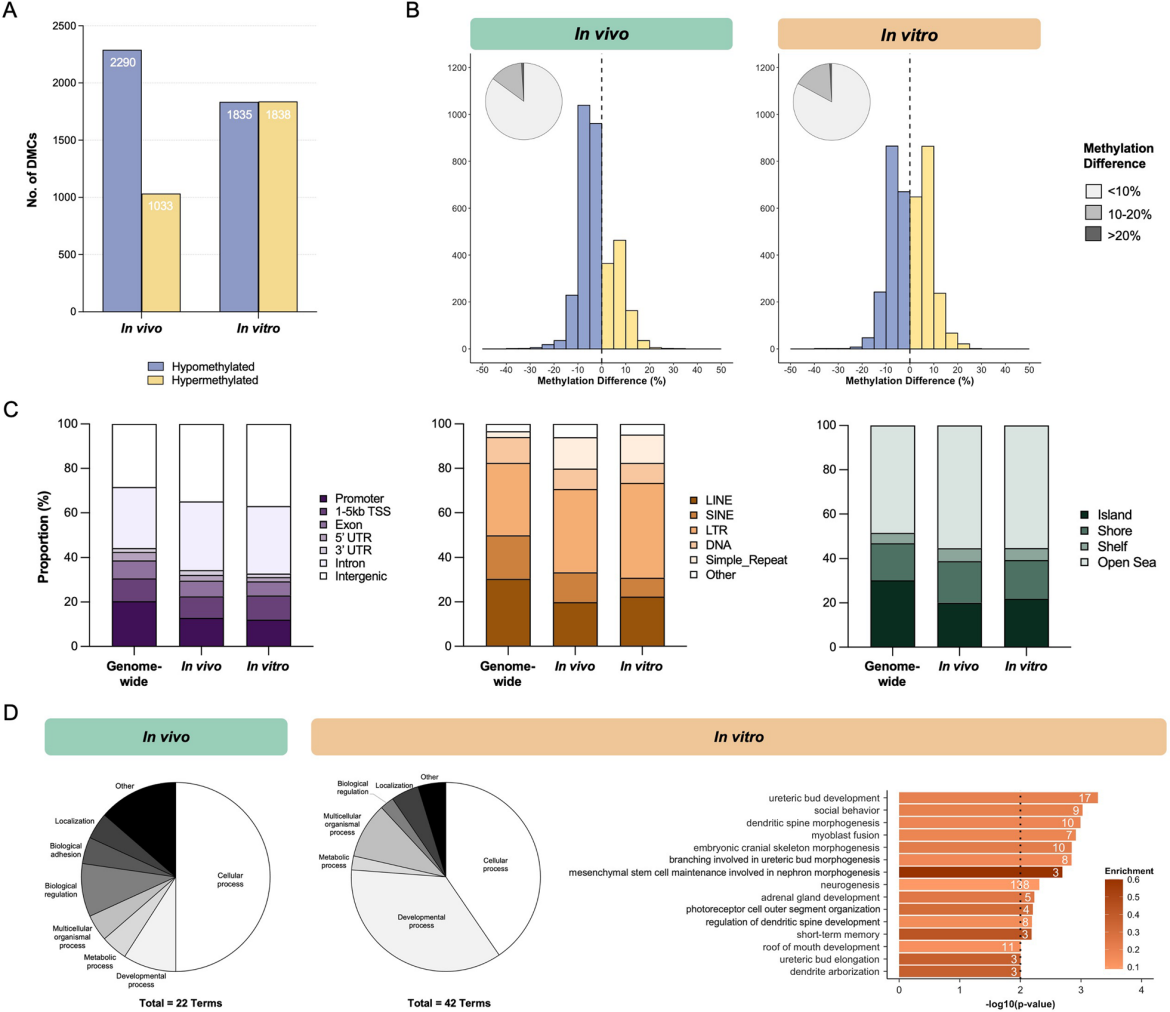
In vivo and in vitro ARTs have distinct effects on genome-wide DNA methylation in cord blood. **A** Total number of hypomethylated and hypermethylated DMCs between in vivo/in *vitro* and control groups. **B** Methylation differences at DMCs between in vivo/in vitro and control groups, with pie charts summarizing the proportion of DMCs demonstrating less than 10%, between 10 and 20% and greater than 20% methylation differences. **C** Distribution of DMCs within genomic regions (left), repetitive elements (middle) and CpG-rich regions (right) in comparison to all captured sites genome-wide. **D** Pie charts demonstrating the categorical distribution of all significant biological processes enriched within genic DMCs for the in vivo/in vitro versus control analysis. Selected significant biological processes from Gene Ontology analysis are shown for the in vitro versus control analysis. The number of observed genes associated with each term is indicated within bars. Enrichment is defined as the proportion of observed genes compared to the number annotated within the whole dataset and is indicated by bar colour. The dotted line represents the significance threshold: weighted Fisher’s *p* value < 0.01

Although we identified DNA methylation differences between in vivo and in vitro ARTs, this analysis did not provide information on their effects on cord blood epigenetic patterning relative to control cord blood samples. Therefore, genome-wide DNA methylation profiles from the in vivo and in vitro groups were compared to the control group. In vivo and in vitro ARTs induced 3323 and 3673 DMCs, respectively (Fig. 5A). Interestingly, the majority of sites significantly altered by in vivo ARTs (69%) exhibited hypomethylation (2290 hypo- vs. 1033 hypermethylated DMCs; Fig. 5A). In contrast, in vitro ARTs were associated with a relatively equal proportion of hypo- and hypermethylation (1835 hypo- vs. 1838 hypermethylated DMCs; Fig. 5A). The methylation changes associated with in vivo and in vitro ARTs were mostly small in magnitude (< 10%) (Fig. 5B). Furthermore, the sites with altered methylation that were associated with in vivo and in vitro ARTs were distributed similarly in terms of genomic, CpG-rich and repeat regions, with enrichment for intronic and intergenic regions, depletion in CpG islands and a slightly increased proportion of LTRs and simple repeats (Fig. 5C).

Despite these similarities, the genic sites affected by each ART subtype were enriched for genes associated with different biological processes. In particular, a strikingly higher proportion of enriched pathways in development was implicated for in vitro ARTs than for in vivo ARTs (Fig. 5D). Specifically, this group, with more invasive ART, seems to involve genes associated with nervous system development, renal system development, endocrine system development, memory, behaviour, and the development of anatomical structures (Fig. 5D). Genes annotated to hypomethylated and hypermethylated DMCs were assessed using STRING to identify gene network clusters enriched for biological pathways (Additional file 1: Fig. S7). Interestingly, we identified a gene network cluster significantly associated with nervous system development and learning using genes annotated to hypomethylated sites associated with in vitro ARTs (Additional file 1: Fig. S7).

**Fig. 6:**
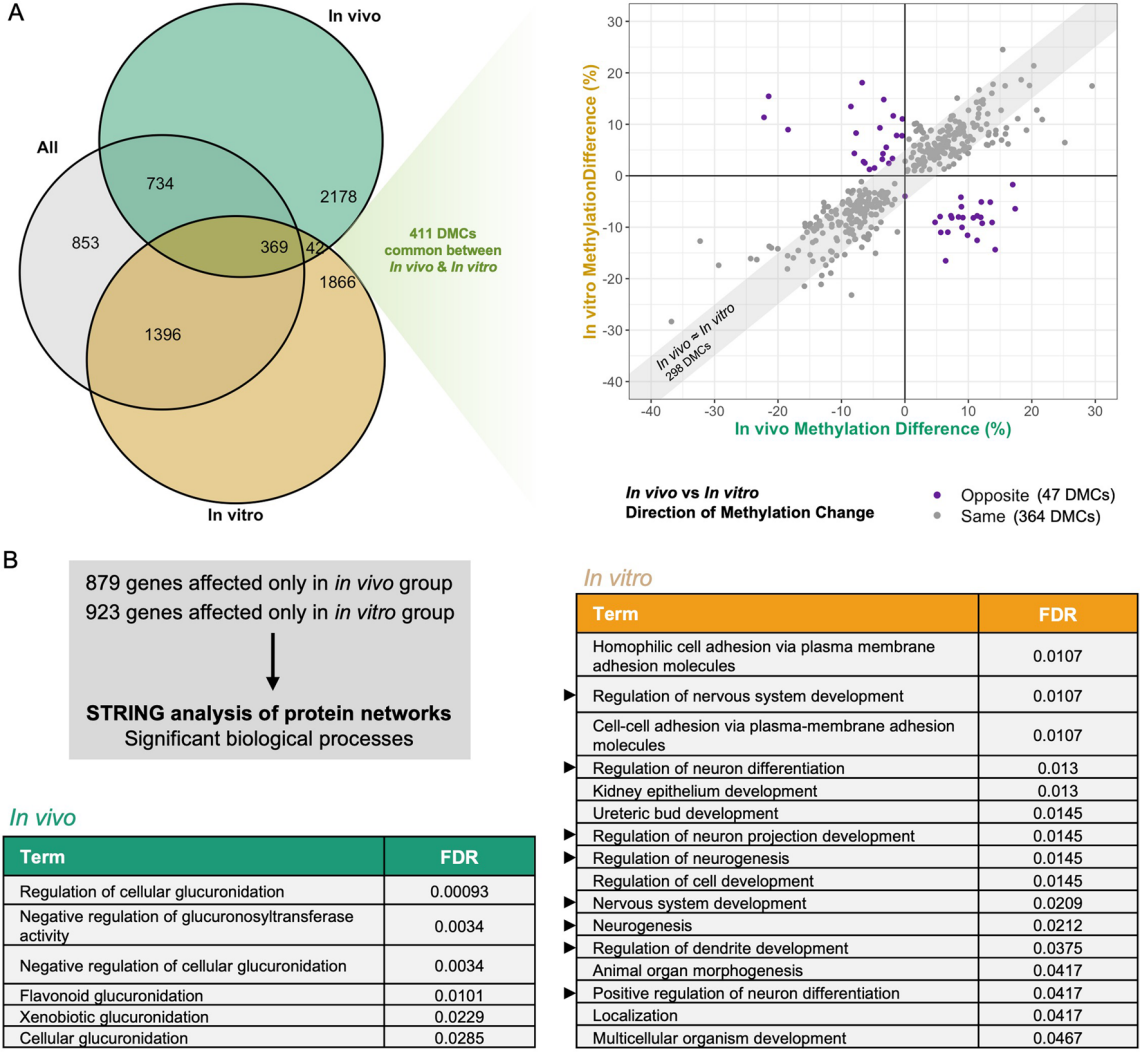
Sites commonly affected by in vivo and in vitro ARTs may represent robust hypofertility-related DNA methylation changes. **A** Venn diagram showing sites commonly affected in both ART subgroups. Methylation differences of in vivo and in vitro groups relative to the control group for DMCs common between both ART subgroups are shown, with DMCs demonstrating methylation changes in the same or opposite direction for both groups indicated in grey and purple, respectively. **B** Significantly enriched biological processes obtained after STRING analysis was performed using genes only annotated to genic DMCs for either the in vivo or in vitro group. Arrows indicate biological processes related to neurodevelopment. FDR; false discovery rate

Next, we determined whether sites commonly affected by in vivo and in vitro ARTs exist. Although both groups vary in terms of the conception methods used to achieve pregnancy, all samples were similar in the fact that they were from individuals with clinically defined infertility. Therefore, these common sites may provide some insight into the effect of underlying parental hypofertility. Interestingly, we found that only 411 sites were altered by in vivo and in vitro ARTs (Fig. 6A). Upon examining the methylation changes associated with each subgroup at these sites, 89% of these sites were similarly affected in terms of their direction of change (364 DMCs), with the majority also similarly affected in terms of magnitude (298 DMCs; Fig. 6A).

To better understand the effect of in vivo and in vitro ARTs, we further examined the genes uniquely associated with each subgroup. In total, 879 and 923 genes were only associated with in vivo or in vitro ARTs, respectively. Using these gene lists, we performed STRING analyses and found that potential protein interactions implicate different biological processes for both ART subtypes; the in vivo group was associated with processes related to glucuronidation, whereas the in vitro group was significantly enriched for processes relating to development, particularly neurodevelopment (Fig. 6B). These results suggest that in vivo and in vitro ARTs may have differing impacts on offspring.

Due to the relatively small number of males and females within the ART/hypofertility subgroups, we did not perform sex-stratified analysis of the effect of in vivo and in vitro ARTs on genome-wide DNA methylation. Nevertheless, when we examined the effect of in vivo and in vitro ARTs relative to the control group for males and females separately at the *KCNQ1OT1* ICR, *H19/IGF2* ICR, *GNAS-NESP* DMR, *GNAS-AS1* DMR, *GNAS-XL* DMR and *GNAS-A/B* DMR, we detected sex-specific effects at certain imprinted loci (refer to Additional file 1: Table S2), particularly at the *KCNQ1OT1* and *H19/IGF2* ICRs (refer to Additional file 1: Fig. S8).

### Merging sites into regions reveals biologically important areas of the genome affected by in vivo and in vitro ARTs

**Fig. 7:**
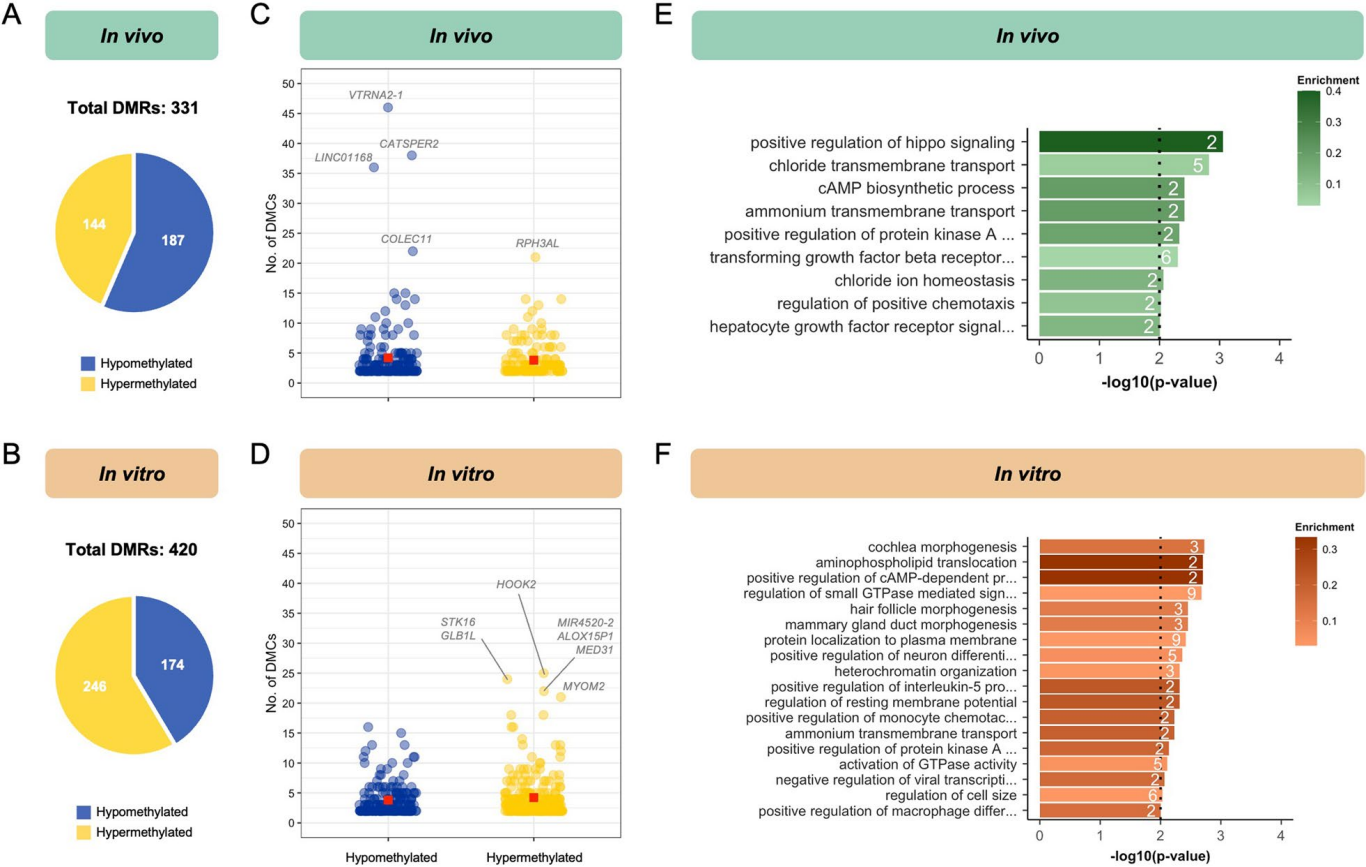
In vivo and in vitro ARTs affect DNA methylation in regions implicated in different biological processes. Number of hypo- and hypermethylated differentially methylated regions (DMRs) in **A** in vivo and **B** in vitro groups relative to the control group. Number of DMCs merged within each hypo- and hypermethylated DMR for **C** in vivo and **D** in vitro groups. Genes annotated to DMRs containing the highest number of DMCs are identified. All significant biological processes from Gene Ontology analysis of all DMRs annotated to genic regions for **E** in vivo and **F** in vitro analyses. The number of observed genes associated with each term is indicated within bars. Enrichment is defined as the proportion of observed genes compared to the number annotated within the whole dataset and is indicated by bar colour. The dotted line represents the significance threshold: weighted Fisher’s *p* value < 0.01

Neighbouring DMCs were merged as previously described to identify regions of differential methylation. Although 60% of DMCs altered by in vivo ARTs (1504 hypo- and 485 hypermethylated sites; Additional file 1: Fig. S2E, F left) remained as individual CpG sites, we identified 187 hypomethylated (mean size 105 bp) and 144 hypermethylated DMRs (mean size 83 bp) (Fig. 7A, Additional file 1: Fig. S2E, F right). In contrast, 54% of DMCs altered by in vitro ARTs (1174 hypo- and 800 hypermethylated sites; Additional file 1: Fig. S2G, H left) remained as individual CpG sites, resulting in 174 hypomethylated (mean size 103 bp) and 246 hypermethylated DMRs (mean size 109 bp) (Fig. 7B; Additional file 1: Fig. S2G, H right). Therefore, in total, more regions were affected by in vitro (420 DMRs, Fig. 7B) than by in vivo (331 DMRs, Fig. 7A) ARTs.

Although the average number of DMCs within a region was relatively small (< 5 DMCs/hypo- and hypermethylated DMR), we identified a number of DMRs highly affected by each ART subtype with > 20 DMCs (Fig. 7C, D). In the case of in vivo ARTs, these regions are annotated to the *VTRNA2-1*, *CATSPER2*, *LINC01168*, *COLEC11*, and *RPH3AL* genes (Fig. 7C), whereas the DMRs highly altered due to in vitro ARTs are annotated to the *HOOK2*, *STK16*, *GLB1L*, *MIR4520-2*, *ALOX15P1*, *MED31* and *MYOM2* genes (Fig. 7D). Gene Ontology enrichment analyses were performed utilizing genic DMRs, with a higher number of significant pathways associated with the use of in vitro ARTs (Fig. 7F) compared to in vivo ARTs (Fig. 7E). Taken together, these data suggest that different ART methods have unique biological implications for offspring.

### Environmentally sensitive sites are vulnerable to ART/hypofertility

**Fig. 8:**
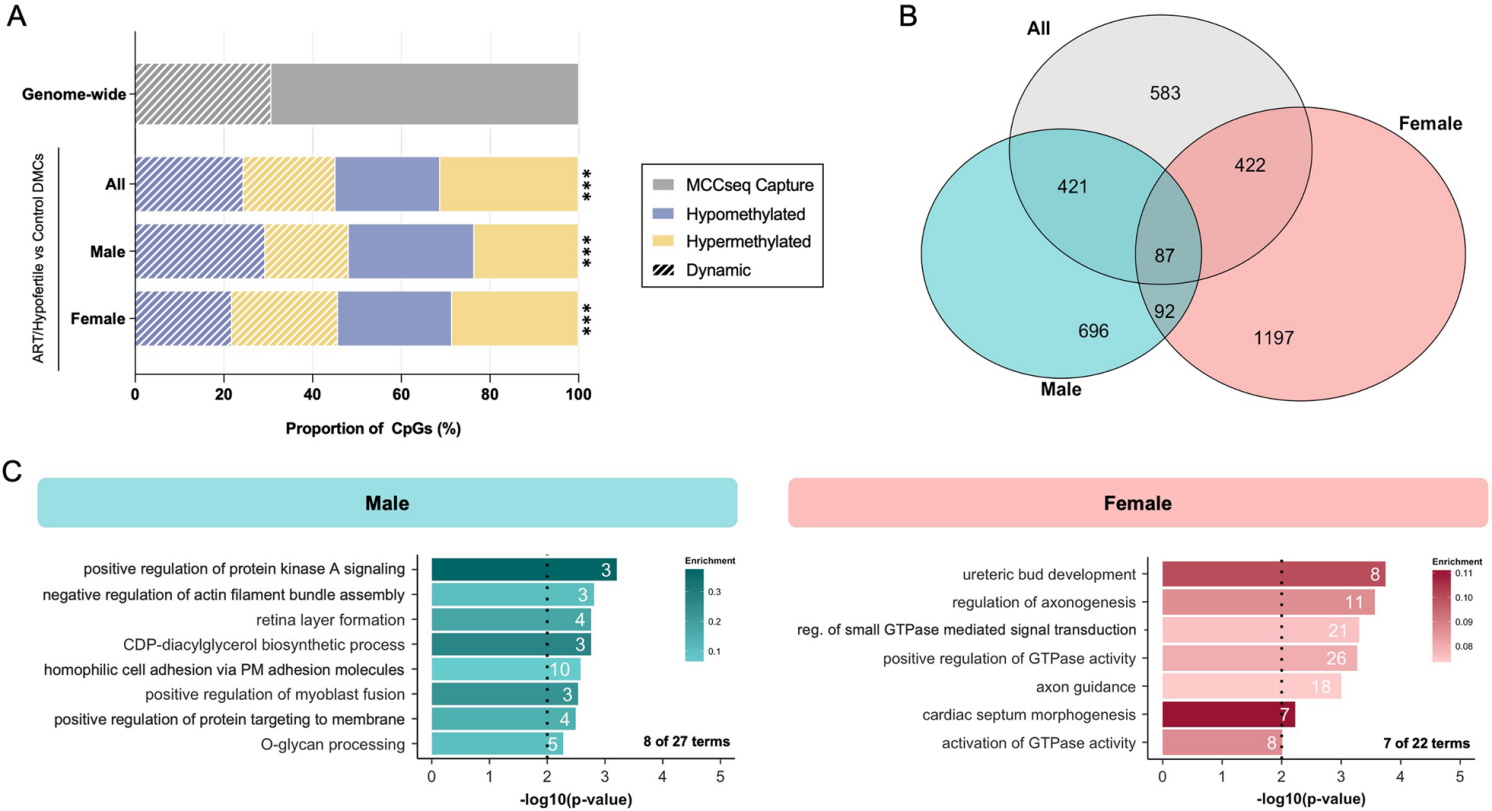
Dynamic sites in the genome are susceptible to ART/hypofertility-induced methylation defects. **A** Proportion of environmentally sensitive dynamic sites among DMCs between ART and control groups when all, only male and only female samples were used for comparison. Chi-square with Yate’s correction was used to compare the proportion of dynamic sites among DMCs relative to all captured sites genome-wide; ***p < 0.001. **B** Venn diagram exclusively showing dynamic DMCs commonly affected by ART using all, only male and only female samples. **C** Significant biological processes from Gene Ontology analysis on all dynamic DMCs annotated to genic regions for the ART versus Control analysis in males and females. The selected biological processes shown were also significantly enriched when all DMCs (dynamic and non-dynamic) were included in the analysis. The number of observed genes associated with each term is indicated within bars. Enrichment is defined as the proportion of observed genes compared to the number annotated within the whole dataset and is indicated by bar colour. The dotted line represents the significance threshold: weighted Fisher’s *p* value < 0.01

MCC-seq covers regions of variable and/or dynamic DNA methylation in sperm that are proven to be environmentally sensitive [14]. Therefore, we determined whether these dynamic sites are also sensitive to ART/hypofertility in cord blood. Interestingly, compared to the background of the MCC-seq, we observed enrichment of dynamic sites altered by ART/hypofertility both in our combined and sex-stratified analyses (*p* < 0.001; Fig. 8A). For all three analyses, a larger density of ART/hypofertility-associated dynamic DMCs exhibited small methylation changes compared to non-dynamic DMCs (Additional file 1: Fig. S9). Although a nearly identical number of dynamic DMCs from our combined analysis were common to our male and female analyses (508 and 509 dynamic DMCs, respectively), only 179 dynamic CpGs were altered in both males and females (Fig. 8B), thus reinforcing the sex-specific manner in which ART/hypofertility may affect the cord blood DNA methylome. Overall, the dynamic DMCs in males and females were significantly enriched for a number of biological processes. Notably, several of these significantly enriched pathways were previously identified in the Gene Ontology analyses performed using all DMCs (these pathways are shown in Fig. 8C). We also examined dynamic sites in DMCs associated with in vivo and in vitro ART subgroups. Despite no difference in the proportion of dynamic sites among DMCs associated with in vitro ARTs when compared to the background of the MCC-seq, we did observe a decreased proportion among in vivo ART-associated DMCs (Additional file 1: Fig. S10). These data suggest that sites sensitive to environmental factors may also be susceptible to disturbance by ART/hypofertility.

## Discussion

There have been concerns regarding the safety of ARTs, as studies have shown that offspring conceived using these methods are at an increased risk for adverse health outcomes [2]. The coincidental timing of ARTs and epigenetic reprogramming events point to ART-induced epigenetic instability contributing to these health risks in ART-conceived populations. For this reason, it is important to characterize and compare epigenetic profiles between ART and naturally conceived infants. Recent studies have adopted epigenome-wide approaches to examine the association between DNA methylation defects and ART; however, reports to date differ immensely from one another in terms of the number of significantly altered sites. For instance, El Hajj et al. [16] identified more than 4000 CpG sites significantly associated with ICSI, whereas other studies reported minimal changes at the CpG level [17, 18].

To our knowledge, this study is the first to utilize a high-throughput MCC-seq approach to explore the epigenetic impact of ART/hypofertility on newborns. Unlike previously used epigenome-wide techniques, this panel provides high coverage sequencing-based information to capture millions of CpGs accurately. Using this method, we compared genome-wide cord blood DNA methylation between 37 ART/hypofertility and 36 control newborns and identified striking sex-specific effects of ART/hypofertility both genome-wide and at imprinting control regions. In addition, we report that in vivo and in vitro ARTs have distinct effects on the epigenome, with more invasive techniques targeting sites implicated in developmental processes.

In our study, we identified 3352 CpG sites that exhibited significant differential methylation between ART/hypofertile and control groups, the majority of which were subtle (< 10% difference between the ART/hypofertile and control groups). Similarly, a study that utilized the Illumina 450 K array to compare ICSI and control cord blood samples found that the DNA methylation defects associated with assisted conception were small in magnitude [16]. There is evidence that environmental factors associated with methylation changes of small effect size, namely, nutritional exposures, can have long-term phenotypic consequences in offspring, as reviewed by El Hajj et al*.* [19]. Therefore, these subtle ART/hypofertility-induced epigenetic abnormalities may have a cumulative effect to potentially influence offspring health.

Our group’s previous study assessing the association between DNA methylation abnormalities and ART/infertility in placentas from singleton pregnancies reported enrichment of abnormal (or “outlier”) DNA methylation profiles among placentas of female infants [8]. This finding suggests the effect of ART/infertility on epigenetic patterning being modulated by infant sex, with females potentially being more susceptible to epigenetic insults by ART/infertility. Accordingly, in the current study we performed sex-stratified analyses to determine whether ART/hypofertility differentially affects male and female infant cord blood. Interestingly, our analyses identified more CpGs significantly affected by assisted conception/hypofertility in female than in male offspring, supporting the idea that females are more epigenetically sensitive to ART/hypofertility.

Alterations associated with ART/hypofertility in males were found to be enriched for metabolic processes, mainly involving carbohydrates and carbohydrate derivatives. This is in line with a recent animal study that demonstrated a more severe metabolic phenotype (elevated insulin, triglycerides, and body fat percentage) induced by IVF in male offspring [20]. ARTs affect glucose homeostasis in male mice [21, 22]. Conversely, our study demonstrated developmental processes, particularly those involved in nervous system and circulatory system development, to be enriched among ART/hypofertility-associated sites in female infant cord blood. Although evidence is limited, it has been suggested that ART confers an increased risk for neurodevelopmental disorders and cardiovascular dysfunction [2]. Taken together, these results suggest that male and female ART-conceived offspring may be susceptible to different diseases or phenotypes later in life.

In our study, the sex-specific nature of ART/hypofertility effects was further supported by the fact that only a small percentage of DMCs were common between sexes, with shared DMCs being differentially affected by ART/hypofertility (i.e. opposite DNA methylation changes are induced). This result is in line with a recent study that reports sex-specific effects of embryo cryopreservation on the placenta in both humans and mice [23]. Not only may our findings explain why the magnitude of methylation changes in the DMCs from combined analysis were smaller, but they may also explain why studies report inconsistent results and relatively minor effects of ART, as most ART studies utilize cohorts consisting of both males and females. These findings suggest the importance of sex-stratified analyses to ensure that significant sex-specific effects of ART/hypofertility are identified.

Despite these striking sex-specific effects, we found some critical genes to be commonly affected by assisted reproduction in males and females. Notably, we reported that DNA methylation at *GLI2* is affected by ART/hypofertility. *GLI2* encodes the zinc finger protein GLI2, a transcription factor that plays an important role in several developmental processes during embryogenesis. Interestingly, a recent study that compared the cord blood DNA methylome of 962 ART-conceived and 983 naturally conceived singletons using the Infinium MethylationEPIC BeadChip identified 176 known genes associated with ART, one of which was *GLI2* [24].

Imprinted genes are implicated in critical processes related to growth, placental function, and neurodevelopment [25]. By using rodent models, epigenetic defects at imprinted genes in tissues from ART pregnancies have been identified [5, 6, 26–28], which was also reported in a number of human ART studies that utilized targeted methylation techniques, as reviewed by Barberet et al*.* [4]. In fact, imprinting methylation errors were found at high frequency even at the early stages of embryogenesis, specifically in Day 3 and blastocyst stage human preimplantation embryos [29]. Our findings presented herein suggest sex-specific impacts of ART/hypofertility on DNA methylation at *KCNQ1OT1*, *H19/IGF2*, *GNAS-NESP* and *GNAS-A/B* ICRs. Indeed, ART/hypofertility was associated with DNA hypomethylation in female infant cord blood at all four regions. Similarly, decreases in ART/infertility-induced methylation at imprinted loci were more prevalent in placentas of females from this same cohort [8]. In addition, female-biased hypomethylation was induced by ART at the *Kcnq1ot1*, *H19* and *Snrpn* ICRs in a mouse model [30]. Because X chromosome inactivation in female preimplantation embryos involves DNA methylation, it has been postulated that females are more susceptible to DNA hypomethylation when specific conditions impede DNA methylation events [31, 32]. Overall, these results reveal that sex-specific ART/infertility effects extend to imprinted loci.

A potential explanation for the inconsistent reports regarding the epigenetic effect of ARTs in human studies is the existence of “epigenetic outliers”, a small group of individuals with early epigenetic patterning that is sensitive to environmental insults such as ART [12]. Although our recent work examining the effect of assisted conception/infertility on the human placenta supports this hypothesis [8], the differences we identified between ART/hypofertility and control cord blood were not driven by outliers. In other words, abnormal (or “outlier”) DNA methylation patterning in cord blood was not associated with the conception method or sex. Animal studies have clearly demonstrated that the placenta is more susceptible to ART-induced DNA methylation defects than the embryo [6, 26, 30]. Therefore, our findings suggest that the placental methylome may be a more sensitive indicator to identify offspring at risk for adverse health outcomes than the cord blood methylome.

In human studies, it is often complicated to discern whether outcomes observed in ART populations are attributable to ARTs, underlying parental infertility, or their combination. Fortunately, information regarding parental history of infertility/hypofertility and time to pregnancy was collected in the 3D study. Therefore, we were able to address this issue by dividing the ART/hypofertile group into subgroups based on whether conception occurred in vivo or in vitro. The in vivo group consisted of samples from subjects with clinically defined infertility who either conceived spontaneously or using IUI; therefore, any discriminating variables between this group and the control group were associated with hypofertility. In contrast, differential methylation between the in vitro and control groups was linked to the combined effect of ART and hypofertility, as the in vitro group included samples from pregnancies conceived using invasive procedures (IVF or ICSI). Here, we report marked differences between in vivo and in vitro subgroups, with the significantly enriched pathways among in vitro genic DMCs involved predominantly in nervous system development, behaviour and memory. Similar to what was reported in a mouse study [32], our results suggest that the developmental consequences of hypofertility may be exacerbated by ARTs through neurodevelopment effects.

In our study, genome-wide methylation was profiled using MCC-seq, which targets significantly more sites than the more commonly used Illumina arrays. This technique targets enhancer and regulatory regions, with no bias towards CpG dense regions. MCC-seq is not only able to accurately detect small methylation differences but also evaluates sites of dynamic methylation proven to be environmentally sensitive in sperm [14, 33]. Here, we report enrichment in the proportion of dynamic sites among DMCs associated with ART/hypofertility. Considering that ARTs involve manipulations that can alter the gametic and/or embryonic environment, our data suggest that these environmentally sensitive sites are also susceptible to ART-induced epigenetic defects. Interestingly, within the ART/hypofertile subgroups, we observed a significantly higher proportion of dynamic sites among in vitro DMCs than among in vivo DMCs. This lends support to our previous conclusion: invasive procedures represent a more harmful environmental exposure for gametes and/or developing embryos.

In addition to the strengths of our study, we recognize that there are also limitations, particularly with regard to the small number of samples. Although we were able to identify differences between in vivo and in vitro ARTs, larger studies are warranted to examine both individual ART techniques and individual aetiologies of hypofertility to ascertain their impact on the cord blood epigenome. In addition, despite having demonstrated that a large majority of DMCs are common between analyses performed with and without the inclusion of pregnancy complications (prematurity, gestational diabetes, pre-eclampsia) as a covariate, we cannot rule out that there may be some effects of pregnancy complications as studies have reported an association with cord blood DNA methylation [34, 35]. As previously mentioned, to our knowledge, our study represents the most comprehensive profiling of DNA methylation changes associated with ART/hypofertility in human cord blood. Nonetheless, we examined only a fraction of the total CpGs that exist in the human genome. Therefore, it is important to consider that for this reason, we may not have gained other important information, such as that for key enhancers and gene control elements.

Despite these limitations, we demonstrate the importance of utilizing comprehensive DNA methylation profiling techniques that are sequencing based because they are quantitative in nature, as opposed to array techniques. Future research is warranted to determine whether DNA methylation perturbations in cord blood associated with ART/hypofertility persist in blood later in life or whether they are corrected. Imprinting control regions are of particular interest because altered DNA methylation at such sites is unlikely to be corrected later in life. Our study also demonstrates the importance of sex stratification in human ART studies. We report opposite DNA methylation changes associated with ART/hypofertility in male and female infants, and future studies are needed to determine whether there are also different clinical effects that can be identified during childhood. In addition, we demonstrate that more invasive ART techniques are associated with potentially more biologically significant DNA methylation changes, suggesting the importance of future studies to assess the safety of these invasive ARTs.

## Conclusion

In our study, we comprehensively profiled the effect of ART/hypofertility on the cord blood DNA methylome utilizing a high-throughput sequencing-based technique. We identified evidence of sex-specific effects of assisted reproduction, with ART/hypofertility being associated with DNA methylation defects at genes related to developmental pathways in females. Overall, these findings highlight the importance of sex stratification in future ART studies. Moreover, our study design allowed us to explore the differential effect of hypofertility and the additional impact of ART. We identified that in vitro ARTs (i.e. more invasive techniques) potentially impact neurodevelopmental processes and target environmentally sensitive sites in the genome.

## Methods

### Participant recruitment and study design

The samples utilized in this study were collected as part of the Quebec-based 3D longitudinal pregnancy cohort study [13]. The 3D study recruited couples between May 2010 and August 2012 at nine different hospitals in Quebec with the main objectives of addressing intrauterine determinants of adverse obstetrical outcomes and links between pregnancy exposures and patterns of early childhood development. A total of 2366 participants in their first trimester of pregnancy were recruited into this study. Importantly, 272 of the recruited couples were clinically diagnosed with infertility and/or utilized assisted reproduction for conception. The breakdown of these 272 subjects is shown in Additional file 1: Fig. S11A. For these participants, questionnaires were completed to gather information about ART treatments. From recruitment to 2 years post-partum, a wide range of information was collected, including medical, obstetrical, environmental and socio-demographic data. Current intravenous drug use, severe illnesses/life-threatening conditions and multiple gestation pregnancies were selected as exclusion criteria for the 3D study. Although several biological specimens were collected at delivery, our epigenetics study examined cord blood samples.

The samples from the 3D study cohort that were part of this cord blood epigenetics study were of one of two groups: control (*n* = 36) or ART/hypofertile (*n* = 37); hypofertile is defined as couples with diagnosed infertility, but who conceived spontaneously (no treatments received) after > 1 year of unprotected intercourse. These 73 samples comprised a subset of the 88 samples (44 control, 44 ART/infertile or hypofertile) examined in our previous placenta epigenetics study [8] chosen from among available cord blood samples. The breakdown of samples used in the current and previous studies is shown in Additional file 1: Fig. S11B. For the original study [8], participants in the control group were matched to those in the ART/hypofertile group based on the following criteria: same delivery hospital, maternal age (± 2 years) at the time of delivery, gestational age at delivery with a matching on prematurity (before or after 37 weeks of gestation and overall ± 2 weeks), baby’s sex, and maternal tobacco use status.

The control group consisted of pregnancies in which natural conception was achieved in < 6 months. The ART/hypofertile group comprised pregnancies with (1) an infertility diagnosis and spontaneous conception after > 1 year of unprotected intercourse or (2) infertility and conception with the use of ART (ovarian stimulation and IUI; IVF; ICSI; culture to blastocyst for IVF and ICSI groups). Pregnancies with spontaneous conception after 6–12 months of unprotected intercourse were not included in this study. Pregnancies with low birth weight, macrosomia, use of preimplantation genetic diagnosis and use of donor sperm were excluded from both the control and ART/hypofertile groups. More information about the control and ART/hypofertile groups is shown in Table 1. For additional analyses, the ART/hypofertile group was separated into subgroups based on whether conception was achieved in vivo (*n* = 17) or in vitro (*n* = 20) (refer to Table 2 for details). The approximate breakdown into in vivo and in vitro groups was originally chosen (for the Choufani et al*.* study [8]) to reflect the breakdown in the overall group of 272 participants and to allow matching with controls.

### MethylC‑capture sequencing (MCC‑seq)

MCC-seq was performed on cord blood DNA samples as previously described [14] by first constructing libraries using KAPA High Throughput Library Preparation Kit (Roche/KAPA Biosystems). Briefly, cord blood DNA (1–2 μg) was spiked with 0.1% (w/w) unmethylated lambda DNA (Promega). The DNA was sonicated (Covaris), and fragment sizes of 300–400 bp were controlled on a Bioanalyzer DNA 1000 Chip (Agilent). Using KAPA Biosystems’ protocols, DNA end repair of double-stranded DNA breaks, 3′-end adenylation, adaptor ligation and clean-up steps were then performed. The sample was bisulfite-converted using the Epitect Fast DNA bisulfite kit (Qiagen) following the manufacturer’s protocol. Following bisulfite conversion, quantification with OliGreen (Life Technology) and amplification with 9–12 PCR cycles using KAPA HiFi HotStart Uracil + DNA Polymerase (Roche/KAPA Biosystems) was performed according to the suggested protocols. The final libraries were purified using Ampure Beads, as validated using Bioanalyzer High Sensitivity DNA Chips (Agilent) and quantified by PicoGreen (Thermo Fisher).

Following library preparations for all individual cord blood samples, regions of interest were captured using the SeqCap Epi Enrichment System protocol (RocheNimbleGen). Equal amounts of multiplexed libraries (84 ng of each; 12 samples per capture) were combined to obtain 1 μg of total input library and hybridized to the capture panel at 47 °C for 72 h. Washing, recovery, PCR amplification of the captured libraries and final purification were conducted following the manufacturer’s recommendations. The quality, concentration and size distribution of the final captured libraries were determined using Bioanalyzer High Sensitivity DNA Chips (Agilent). The capture libraries were sequenced with a 200-cycle S2 kit (100-bp paired-end sequencing) using the NovaSeq 6000 following the NovaSeq XP workflow.

### Sequencing data processing

Targeted MCC-Seq raw reads were processed using the GenPipes pipeline [36]. Specifically, the MCC-Seq paired-end raw reads were trimmed for quality (phred33 ≥ 30) and Illumina adapters using Trimmomatic (version 0.36) [37]. The trimmed reads were aligned to the bisulfite-converted hg19/GRCh37 reference genome using Bismark (version 0.18.2) [38] with Bowtie 2 (version 2.3.1) [39] in paired-end mode with default settings. The aligned BAM files were then de-duplicated using Picard (Broad Institute, version 2.9.0). Methylation calls were extracted using Bismark. BisSNP (version 0.82.2) [40] was run on the de-duplicated BAM files to call variants. CpGs that were found to overlap with SNPs (dbSNP 137), the Data Analysis Center (DAC) Blacklisted Regions or Duke Excluded Regions (generated by the ENCODE project) were removed. CpG sites with < 15 × or > 500× read coverage per sample were also discarded before merging. We then merged all samples to obtain a CpG profile matrix. After that, only CpG sites covered by ≥ 30 samples were retained. In addition, CpGs located on sex chromosomes were further removed for downstream analysis. Cumulative distribution plots for CpG counts by sample size before and after read coverage filtering are shown for all samples, ART/hypofertile group samples and control group samples in Additional file 1: Fig. S12.

### Outlier detection

Samples with “irregular” DNA methylation patterns (relative to other samples), otherwise known as outlier samples, were identified as previously described by Choufani et al*.* [8]. Briefly, cord blood samples were first sorted based on the PC1 value, and the gap sizes between neighbouring samples were computed. Next, a changepoint detection algorithm was performed (R package *changepoint, cpt.mean* function) [41], and a gap size that was significantly different from the others based on “at most one change” (AMOC) was identified. This changepoint gap identified based on this method was deemed to separate outliers from the remaining sample set.

### Statistical analyses and data visualization

Generalized linear regression models (GLMs) were built to assess associations between DNA methylation and ART/hypofertility based on (a) all samples (combined analysis), (b) males only and c) females only. We also utilized this method to explore epigenetic differences between ART subtypes both directly (in vitro vs. in vivo) and indirectly relative to the control (in vivo/in vitro vs. control). All models were adjusted for infant sex, except for sex-specific models. The models were also adjusted by correcting for the proportions of cord blood cell types as estimated using a reference-based method (Additional file 1: Fig. S13). Specifically, cord blood deconvolution was performed using the projectCellType() function in the minfi R package [42] based on an umbilical cord blood reference panel of cell-type-specific CpGs in B cells, CD4+ T cells, CD8+ T cells, granulocytes, monocytes, natural killer cells and nucleated red blood cells [43]. This panel was originally identified using the Illumina Infinium HumanMethylation450 BeadChip array, and overlapping CpGs between the reference panel and the MCC-Seq CpGs were thus extracted. CpGs covered by at least 90% of the samples were further processed, and missing data for each CpG were imputed with the mean methylation level of all other samples at that specific locus. For this, 345 CpGs covering 43–58 CpGs per cell type were used to estimate the cord blood cell-type proportions. The male and female models were only adjusted for cord blood cell-type proportions. Owing to the fact that pregnancy complications (prematurity, gestational diabetes and pre-eclampsia) were found in both the control and ART/hypofertile groups (Table 1), and that these may influence DNA methylation, we carried out an additional analysis adding the presence of any complications as a covariate; this was performed on our main all samples (combined) analysis, as well as our sex-strafied analyses. We used the R function glm (R version 3.6.0) with the binomial family to fit the models and calculated p values for variables of interest. The obtained p values were corrected by generating false discovery proportion q values using the R package *q values* [44]. The significance threshold was set as *q* < 0.05 for all DMC analyses performed (refer to Additional file 3 for the list of significant DMCs for all comparisons). Visualization of the methylation values at all DMCs for each comparison is also provided in Additional file 1: Fig. S14. Merged regions or differentially methylated regions (DMRs) were identified using neighbouring DMCs within 250 bp. DMCs were merged using the merge function of the BEDTools software suite. Only CpGs with the same direction of differential methylation were merged, irrespective of their genomic region (promoter, intron, exon, etc.).

Graphs were created using GraphPad Prism (version 9.3.1) and Rstudio (version 4.1.2). Pie charts were generated using Microsoft Excel (version 16.61.1). Statistical analyses were performed using GraphPad Prism (version 9.3.1), with statistical significance set at *p* < 0.05 unless otherwise indicated. Absolute values were compared by Fisher’s exact test (categorical demographic variables, normal vs. outlier proportions) or Chi-square test with Yates’ correction (CpG/DMC distributions among genomic, CpG-rich and repeat regions, and proportion of dynamic CpGs). Unpaired t tests were performed to compare numerical demographic variables. Two-way ANOVA with Bonferroni correction was used to compare overall DNA methylation levels at ICRs between ART/hypofertile and control groups for males and females.

Annotation based on genomic regions and CpG-rich regions was performed using the R package *annotatr* (version 1.18.1) [45]. Repeat elements were annotated with HOMER using default parameters. Gene Ontology analysis was performed on genic regions using the R package *TopGO* (version 2.44.0) [46] (refer to Additional file 4 for all gene ontology results). Venn diagrams were generated using the R package *VennDiagram* (version 1.7.3) [47]. The heatmap was produced using R package *pheatmap* (version 1.0.12) [48], with clustering based on Manhattan ordering performed using R package *seriation* (version 1.3.5) [49]. All other R-generated plots were produced with *ggplot2* (version 3.3.5). The STRING database website [50] was used for gene interaction network analyses, with k-means clustering performed to generate three clusters (default value) (refer to Additional file 5 for all biological processes significantly associated with clusters).

## Supplementary Information

**Additional file 1.** Supplementary figures and tables.

**Additional file 2.** Information concerning imprinted control regions and their methylation analysis.

**Additional file 3.** List of all significant DMCs for the different comparisons performed.

**Additional file 4.** List of all significant GO terms discovered for the different comparisons performed

**Additional file 5.** List of all biological processes significantly associated with clusters through STRING analysis.

## Data Availability

Sequencing data for the MCC-Seq have been submitted to the European Genome-phenome Archive under study ID: EGAS00001006643 (Data set ID: EGAD0001009495).

## Abbreviations

ART: Assisted reproductive technology
3D: Design, Develop, Discover
IVF: In vitro fertilization
ICSI: Intracytoplasmic sperm injection
MCC-seq: 5-Methylcytosine capture sequencing
BMI: Body mass index
> 1 yr: Spontaneous pregnancy after > 1 year of unprotected intercourse
IUI: Intrauterine insemination
DMCs: Differentially methylated CpGs
TSS: Transcription start site
LTRs: Long terminal repeats
DMRs: Differentially methylated regions
ICRs: Imprinting control regions
PCAs: Principal component analyses
PC1: Principal component 1
AMOC: “At most one change”
GLMs: Generalized linear regression models
