# Supplementary material for: Capturing sex-specific and hypofertility-linked effects of assisted reproductive technologies on the cord blood DNA methylome
Source: Clin Epigenetics. 2023 May 11;15:82. doi: 10.1186/s13148-023-01497-7 (PMC10176895; doi:10.1186/s13148-023-01497-7)
Supplement: Supplementary file 1 — Additional file 1. Supplementary figures and tables. [file 13148_2023_1497_MOESM1_ESM.pdf]

Supplementary Information

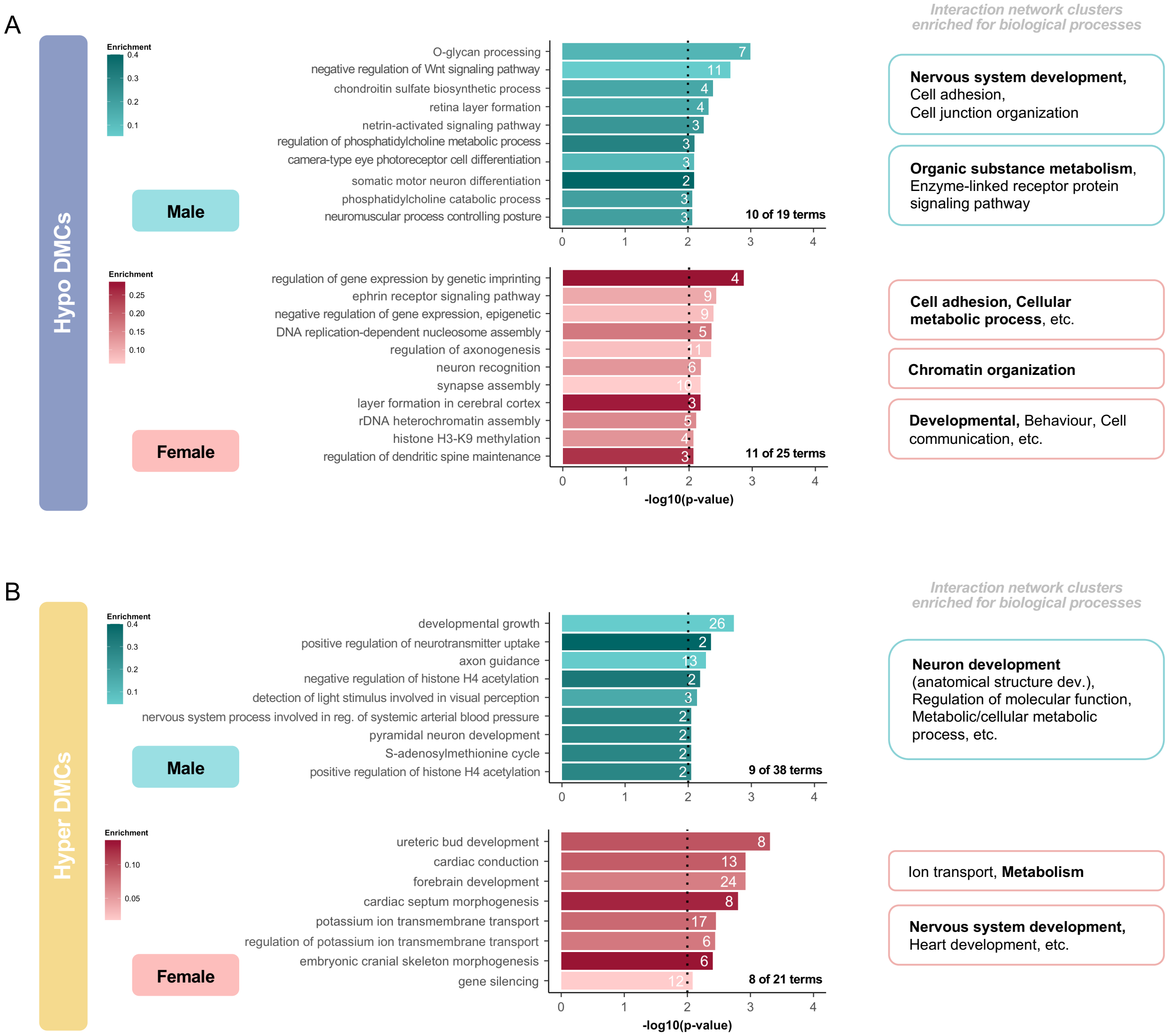

Figure S1: Biological processes enriched in hypomethylated and hypermethylated DMCs between ART/hypofertile and control groups in males and females. Selected significant biological processes from gene ontology analysis on the DMCs located within genic regions with hypomethylation **(A)** and hypermethylation **(B)**. The number of observed genes associated with each term is indicated within the bars. Enrichment is defined as the proportion of observed genes compared to the number annotated within the whole dataset and is indicated by the bar colors. STRING interaction network clusters with significantly enriched biological processes are summarized for each analysis on the right.

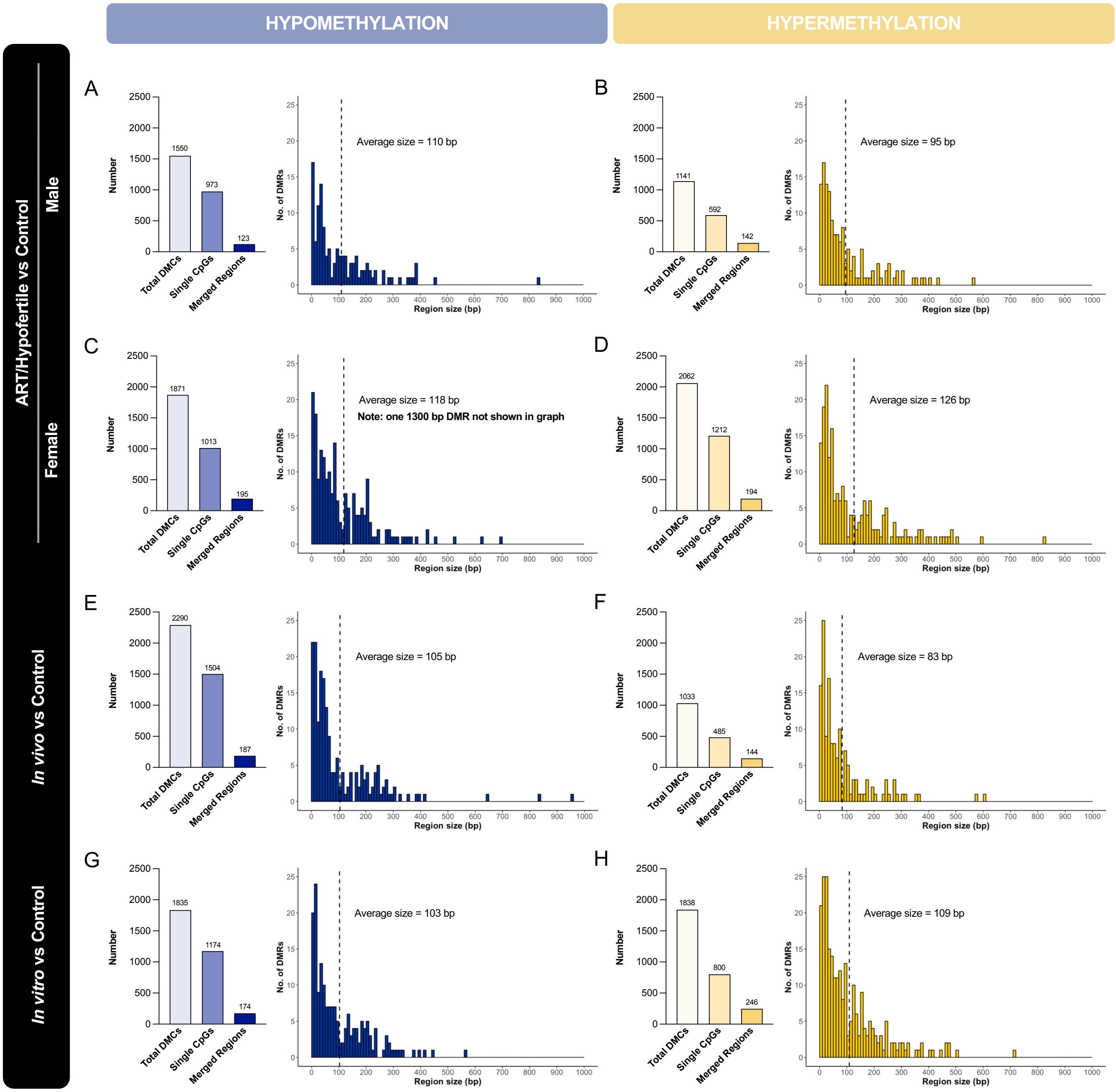

Figure S2: Differentially methylated regions following ART/hypofertility. The effect of ART/hypofertility in males (**A-B**) and females (**C-D**), and the effect of ART/hypofertile subgroups, defined as pregnancies conceived *in vivo* (**E-F**) *and in vitro* (**G-H**). The results of merging hypomethylated and hypermethylated DMCs into regions are shown in blue and yellow, respectively. For each, the first graph depicts the number of total DMCs, isolated single CpGs and merged regions (obtained following the merging of neighboring DMCs within 250 bp from one another), and the second graph shows the distribution of merged region sizes.

Table S1: Imprinting control regions of interest

Imprinting control regions	chr	start	end	Origin	Type	Total no. of CpGs	**CpGs captured[a]**	CpGs on 450K array[a]	CpGs in common[b]
KCNQ1OT1:TSS-DMR	chr11	2719948	2722259	M	Oocyte gDMR	192	**97 (50.5%)**	29 (15.1%)	28
H19/IGF2:IG-DMR	chr11	2018812	2024740	P	Sperm gDMR	250	**106 (42.4%)**	45 (18.0%)	36
GNAS-NESP:TSS-DMR	chr20	57414039	57418612	P	secondary DMR	257	**113 (44.0%)**	23 (8.9%)	22
GNAS-AS1:TSS-DMR	chr20	57425649	57428033	M	Oocyte gDMR	128	**106 (82.8%)**	62 (48.4%)	61
GNAS-XL:Ex1-DMR	chr20	57428905	57431463	M	Oocyte gDMR	200	**65 (32.5%)**	6 (3.0%)	5
GNAS A/B:TSS-DMR	chr20	57463265	57465201	M	secondary DMR	198	**100 (50.5%)**	40 (20.2%)	40

Coordinates, origin and type obtained from Monk D, *et al.* Epigenetics 2018, 13(2):117-121.
TSS, transcription start site; DMR, differentially methylated region; chr, chromosome; M, maternal; P, paternal
[a] N (% compared to Total no. of CpGs)
[b] Number of CpGs covered by both MCC-seq capture and Illumina 450K array

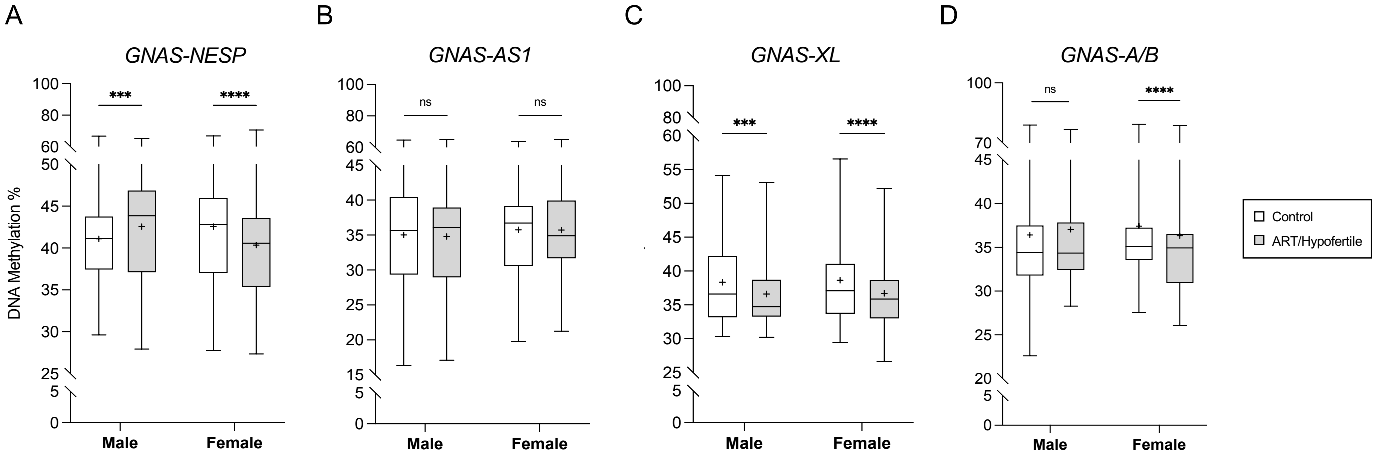

Figure S3: Effect of ART/hypofertility on DNA methylation at *GNAS* DMRs in males and females. DNA methylation was compared between ART/hypofertile and control groups after sex stratification at the paternally methylated *GNAS-NESP* (**A**) DMR and the maternally methylated *GNAS-AS1* (**B**), *GNAS-XL* **(C)** and *GNAS-A/B* (**D**) DMRs. To avoid potential skewing caused by missing data, methylation values were averaged across samples for each CpG. Therefore, average DNA methylation at each individual CpG was used to generate the plot. Box plots bodies extend from the 25th too 75th percentiles with the whiskers extending to the minimum and maximum data values; + represents the mean and the line within the box represents the median. Two-way ANOVA with Bonferroni correction for multiple comparisons was used to compare ART/hypofertile and control groups for males and females; ***p<0.001, ****p<0.0001 and ns signifies no statistically significant difference.

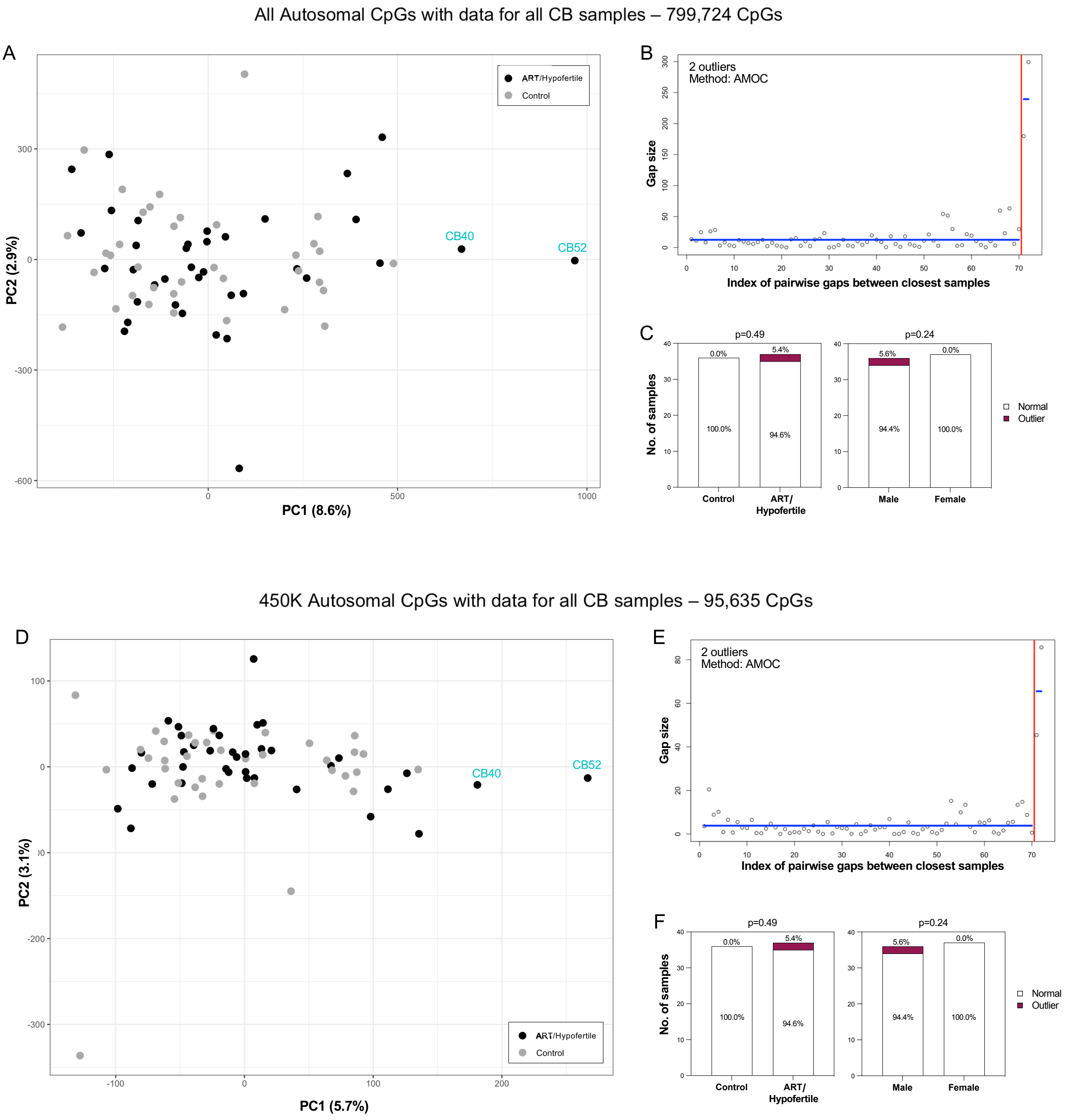

Figure S4: Detection of outlier cord blood samples using CpG sites located in autosomes. Outlier samples were detected using methylation profiles for (**A-C**) all autosomal sites on the MCC-seq capture and (**D-F**) only the autosomal sites targeted by the Illumina 450K array. (**A, D**) PCA was performed using these methylation profiles. Sites with missing data were excluded from the PCA. Outlier sample IDs are shown, with blue and pink colored IDs representing cord blood corresponding to male and female infants, respectively. (**B, E**) Outliers were detected through changepoint detection in mean gap size between cord blood samples along PC1. The "at most one change" (AMOC) method was used to detect the change between normal and outlier mean gap sizes (horizontal lines). Each circle represents an individual gap between a neighboring sample. (**C, F**) The association between outlier status and clinical features (ART/hypofertility status & sex) was examined to determine whether outlier samples are enriched within a specific group and p-values obtained through Fisher's exact test are reported for each comparison.

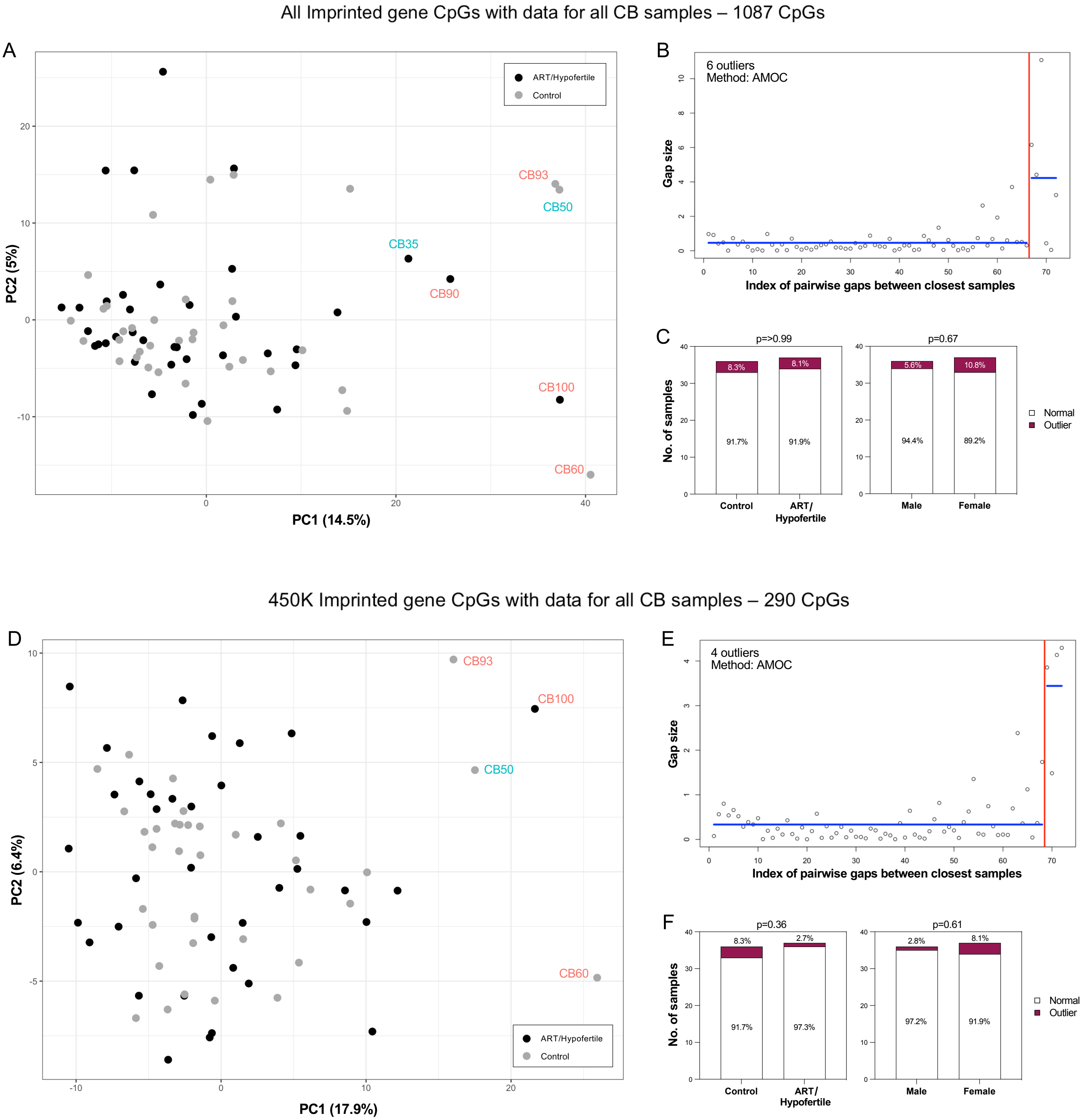

Figure S5: Detection of outlier cord blood samples using CpG sites located in imprinted regions. Outlier samples were detected using methylation profiles for (**A-C**) all ICR sites on the MCC-seq capture and (**D-F**) only the ICR sites targeted by the Illumina 450K array. (**A, D**) PCA was performed using these methylation profiles. Sites with missing data were excluded from the PCA. Outlier sample IDs are shown, with blue and pink colored IDs representing cord blood corresponding to male and female infants, respectively. (**B, E**) Outliers were detected through changepoint detection in mean gap size between cord blood samples along PC1. The ”at most one change” (AMOC) method was used to detect the change between normal and outlier mean gap sizes (horizontal lines). Each circle represents an individual gap between a neighboring sample. (**C, F**) The association between outlier status and clinical features (ART/hypofertility status & sex) was examined to determine whether outlier samples are enriched within a specific group and p-values obtained through Fisher’s exact test are reported for each comparison.

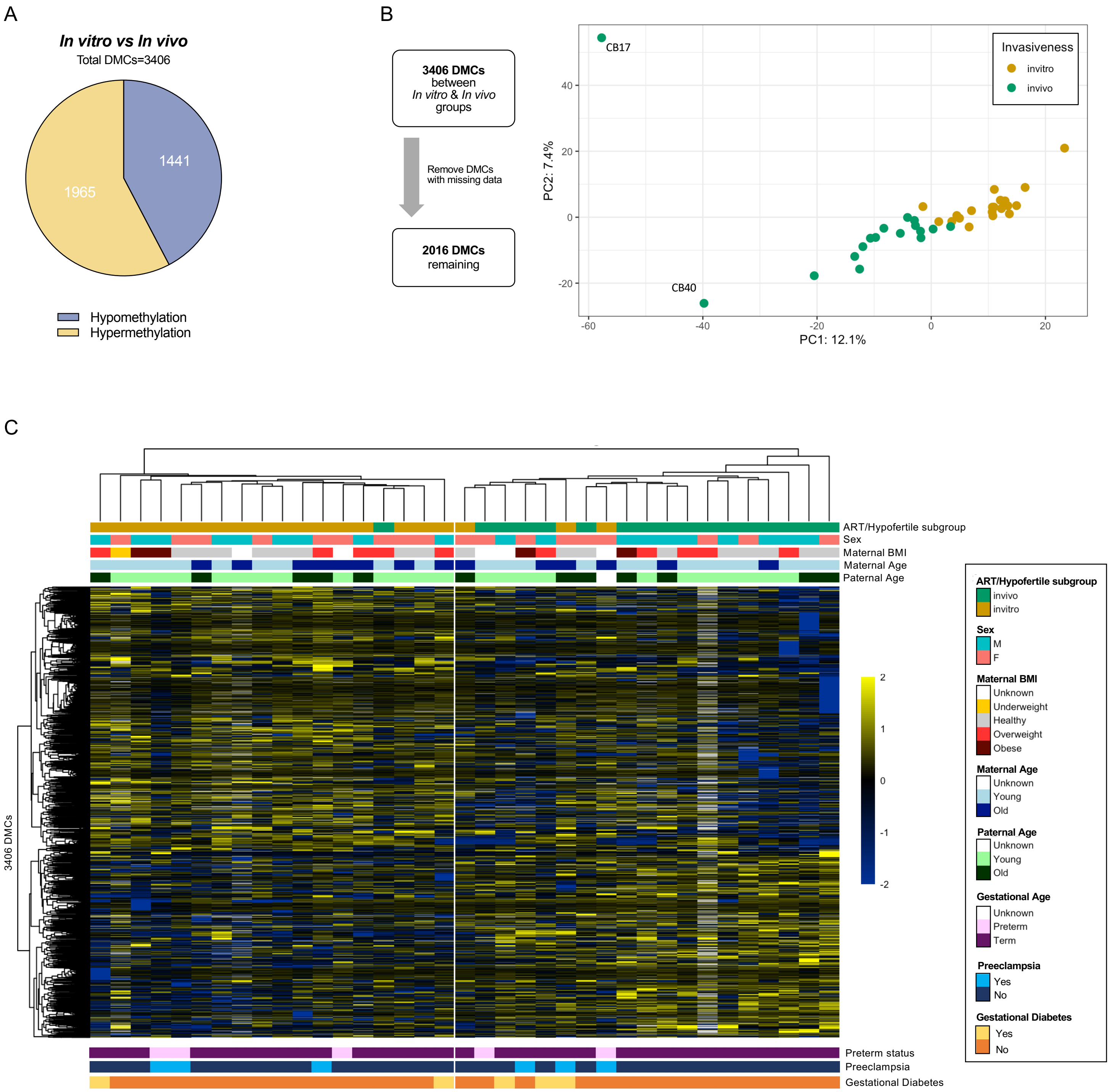

Figure S6: Direct comparison between *in vivo* and *in vitro* subgroups. (A) Total number of hypomethylated and hypermethylated DMCs altered by *in vitro* ARTs relative to *in vivo* ARTs. **(B)** PCA using the DMCs without any missing data. **(C)** Heat map clustering using all DMCs with parental and birth characteristic categories shown.

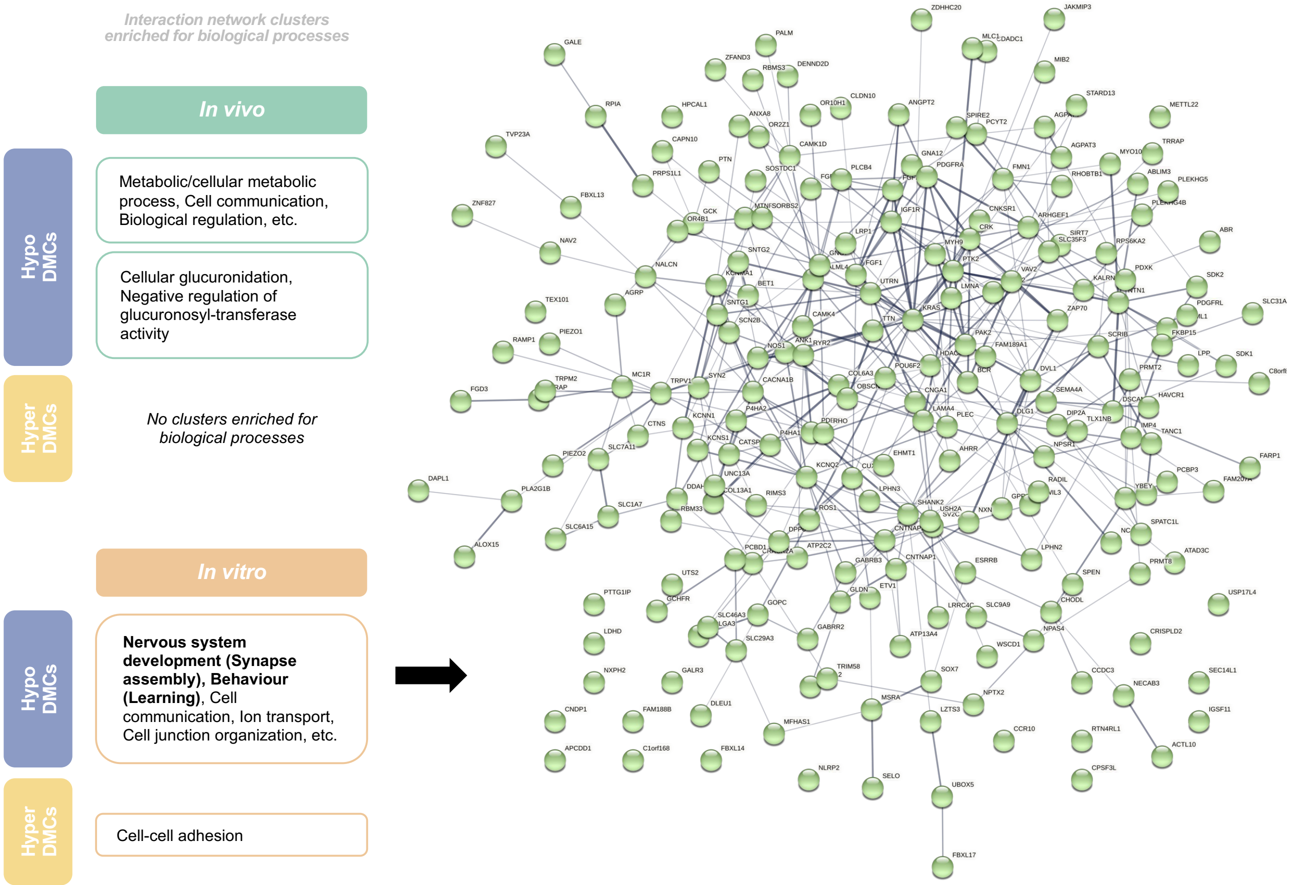

Figure S7: Summary of STRING interaction network clusters with significant enrichment for biological processes. STRING analysis was conducted using genes overlapping hypomethylated and hypermethylated DMCs from *in vivo* and *in vitro* analyses. An interaction network enriched for pathways implicated in nervous system development and behaviour was identified using genes annotated to DMCs hypomethylated in the *in vitro* group relative to the control group.

Table S2: Summary of imprinting control regions results.

Imprinted gene		All	Male	Female
KCNQ1OT1	ART/Hypofertile vs Control	Hyper	Hyper	Hypo
	In vivo vs Control	Hyper	Hyper	ns
	In vitro vs Control	ns	Hyper	Hypo
H19/IGF2	ART/Hypofertile vs Control	Hypo	Hyper	Hypo
	In vivo vs Control	ns	Hyper	Hypo
	In vitro vs Control	Hypo	Hyper	Hypo
GNAS-NESP	ART/Hypofertile vs Control	ns	Hyper	Hypo
	In vivo vs Control	ns	Hyper	Hypo
	In vitro vs Control	Hypo	Hyper	Hypo
GNAS-AS1	ART/Hypofertile vs Control	ns	ns	ns
	In vivo vs Control	Hyper	ns	Hyper
	In vitro vs Control	Hypo	Hypo	ns
GNAS-XL	ART/Hypofertile vs Control	Hypo	Hypo	Hypo
	In vivo vs Control	Hypo	Hypo	ns
	In vitro vs Control	Hypo	ns	Hypo
GNAS-A/B	ART/Hypofertile vs Control	ns	ns	Hypo
	In vivo vs Control	ns	ns	ns
	In vitro vs Control	ns	Hyper	Hypo

ART/Hypofertile (n=37); Control (n=36); *In vivo* (n=17); *In vitro* (n=20); >1yr (n=8); IUI (n=9)

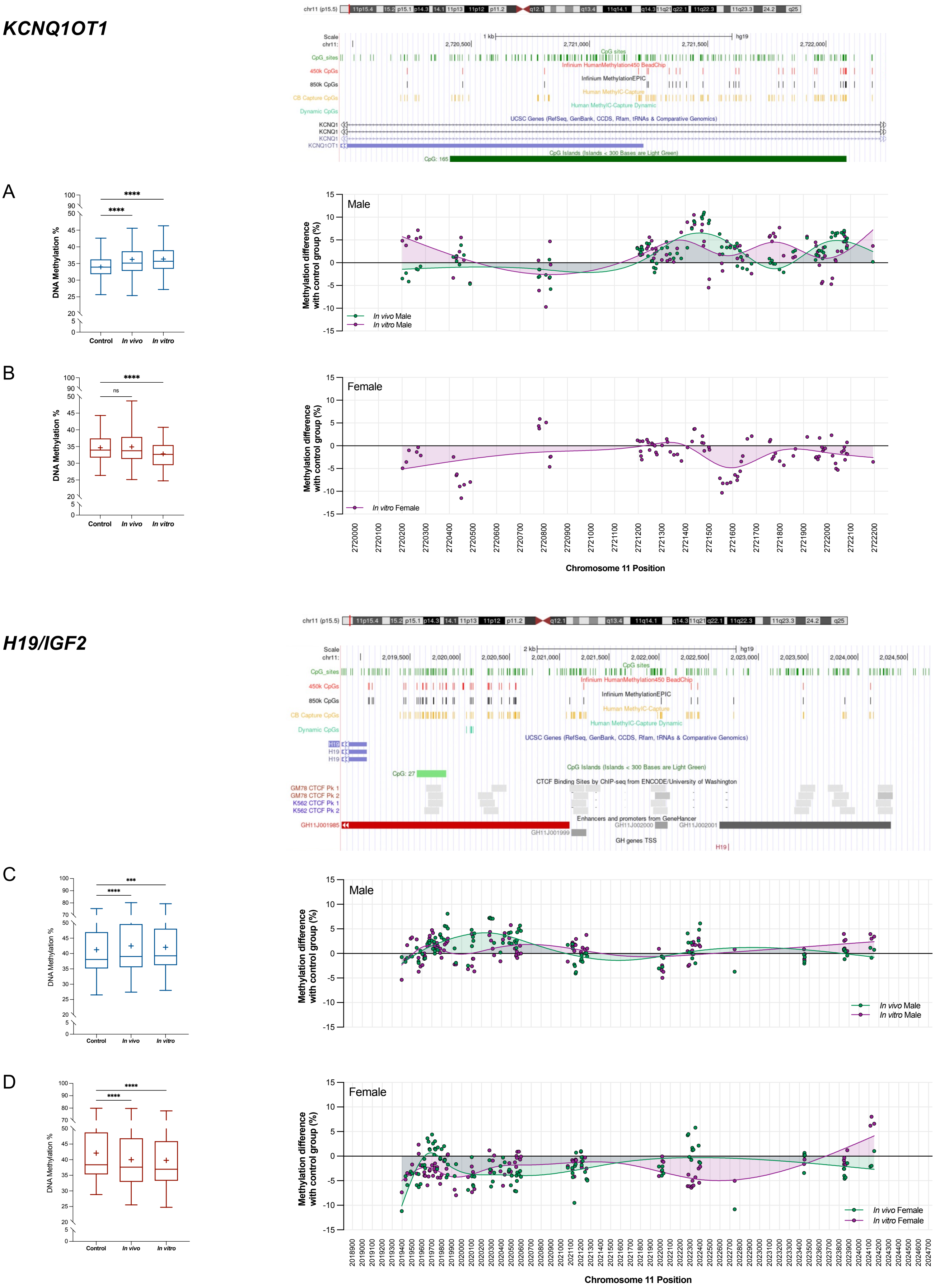

KCNQ1OT1
H19/IGF2
A
B
C
D
DNA Methylation %
Control
In vivo
In vitro
****
***
ns
Male
Female
Methylation difference with control group (%)
In vivo Male
In vitro Male
In vitro Female
In vivo Female
Chromosome 11 Position
chr11 (p15.5)
CpG Islands (Islands < 300 Bases are Light Green)
UCSC Genes (RefSeq, GenBank, CCDS, Rfam, tRNAs & Comparative Genomics)
CTCF Binding Sites by ChIP-seq from ENCODE/University of Washington
Enhancers and promoters from GeneHancer

Figure S8: Sex-specific effect of *in vivo*/*in vitro* ARTs on DNA methylation at imprinting control regions (ICRs). DNA methylation within the maternally methylated *KCNQ1OT1* ICR (97 CpGs) (**A**) and paternally methylated *H19/IGF2* ICR (106 CpGs) (**B**) was compared between *in vivo*/*in vitro* and control groups after sex stratification. To avoid potential skewing caused by missing data, methylation at each CpG was averaged across samples for each CpG. Box plots bodies extend from the 25th to 75th percentiles with the whiskers extending to the minimum and maximum data values; + represents the mean and the line within the box represents the median of all CpGs. For each sex, one-way ANOVA with Bonferroni correction for multiple comparisons was used to compare *in vivo* and *in vitro* groups to the control group; ***p<0.001, ****p<0.0001. UCSC Genome Browser view of the ICR of *KCNQ1OT1* (**C**) and *H19/IGF2* (**D**) with DNA methylation difference between *in vivo*/*in vitro* and control groups shown for each captured CpG. Custom tracks indicate CpG sites (green), CpGs analyzed by the Illumina HumanMethylation450 array (red), CpGs analyzed by the Illumina HumanMethylationEPIC array (black), the sites captured in our study by the MCC-seq capture (gold), and Sperm Dynamic CpG sites (light green). A data point represents the methylation difference between *in vivo*/*in vitro* and control groups at a CpG, which was calculated by averaging all replicates for each group and using the mean values to compute the difference (*In vivo*-Control or *In vitro*-Control). Means values are shown, and each graph contains a smoothing spline curve (using 8 knots) to demonstrate the overall methylation difference trend across the loci.

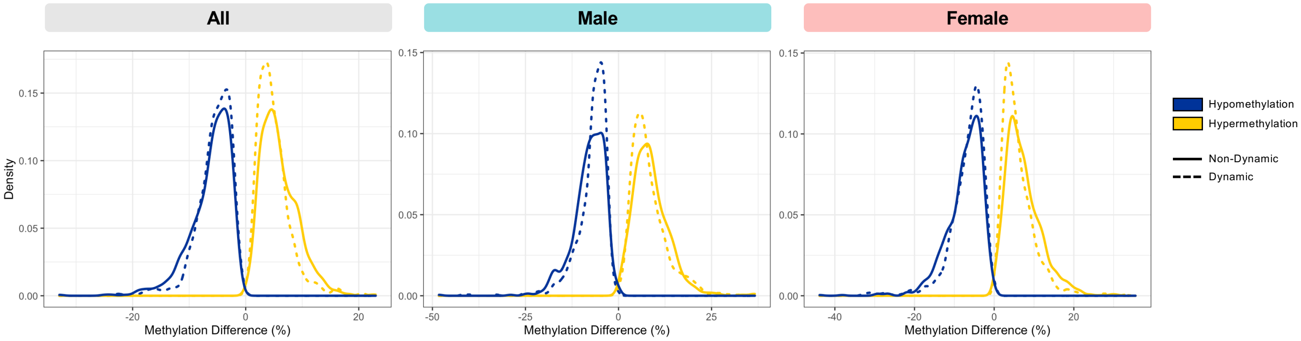

Figure S9: Density plot of methylation differences between ART/hypofertile and control groups at non-dynamic versus dynamic DMCs. Density plots are shown for DMCs identified using all, only male or only female samples.

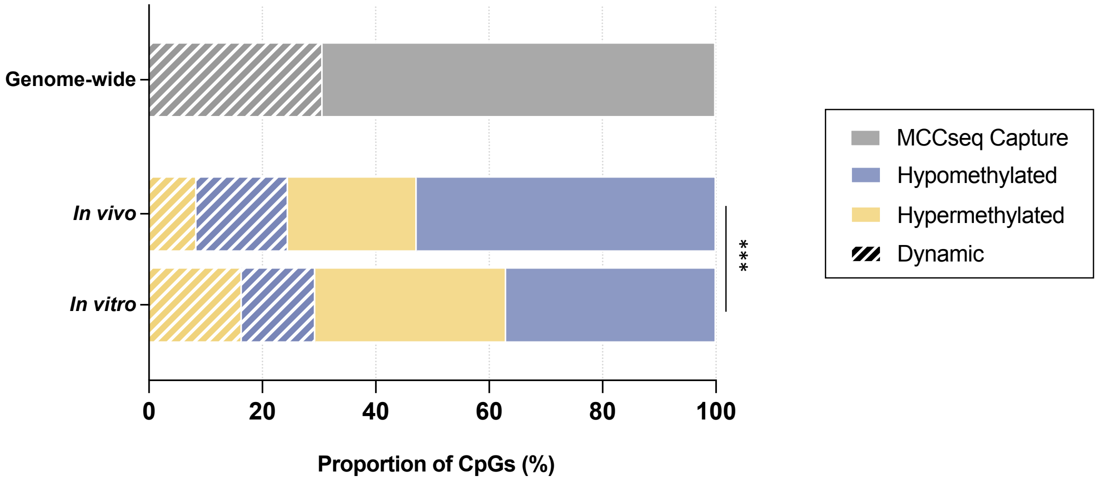

Figure S10: Higher proportion of DMCs affected by *in vitro* ARTs are dynamic compared to *in vivo* ARTs. Proportion of environmentally sensitive dynamic sites among DMCs between *in vivo*/*in vitro* subgroups and control group are shown. Chi-square with Yate's correction was used to compare the proportion of dynamic sites among DMCs between ART/hypofertile subgroups; *** p<0.001.

A

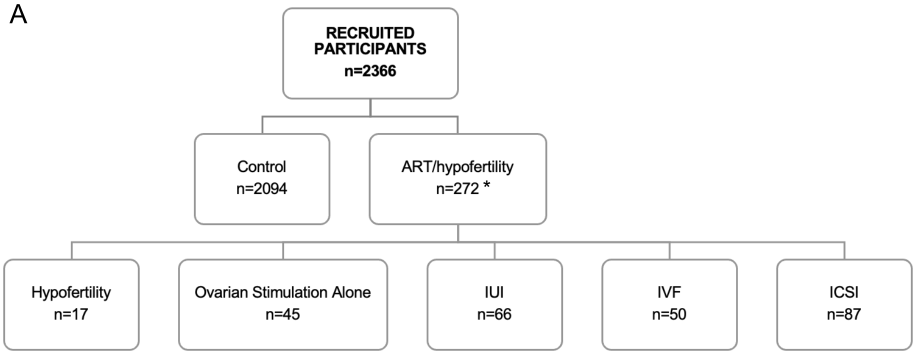

B

	Placenta study[a]	Cord blood study
	n values	
Control	44	36
>1yr	10	8
IUI	11	9
IVF	5	4
ICSI	18	16

[a] Choufani et al. (2019)

Figure S11: Recruitment flow chart and cohort breakdown. (**A**) Flow chart categorizing all participants recruited into the Quebec-based Canadian 3D (Design, Develop, Discover) longitudinal pregnancy cohort. (**B**) The breakdown of the samples used in the previous and current studies examining the effect of ART/hypofertility on the placental and cord blood epigenome, respectively. >1yr, spontaneous pregnancy after >1 year of unprotected intercourse; IUI, intrauterine insemination; IVF, *in vitro* fertilization; ICSI, intracytoplasmic sperm injection. *Note that the ART/hypofertility type was undetermined for 7 of the 272.

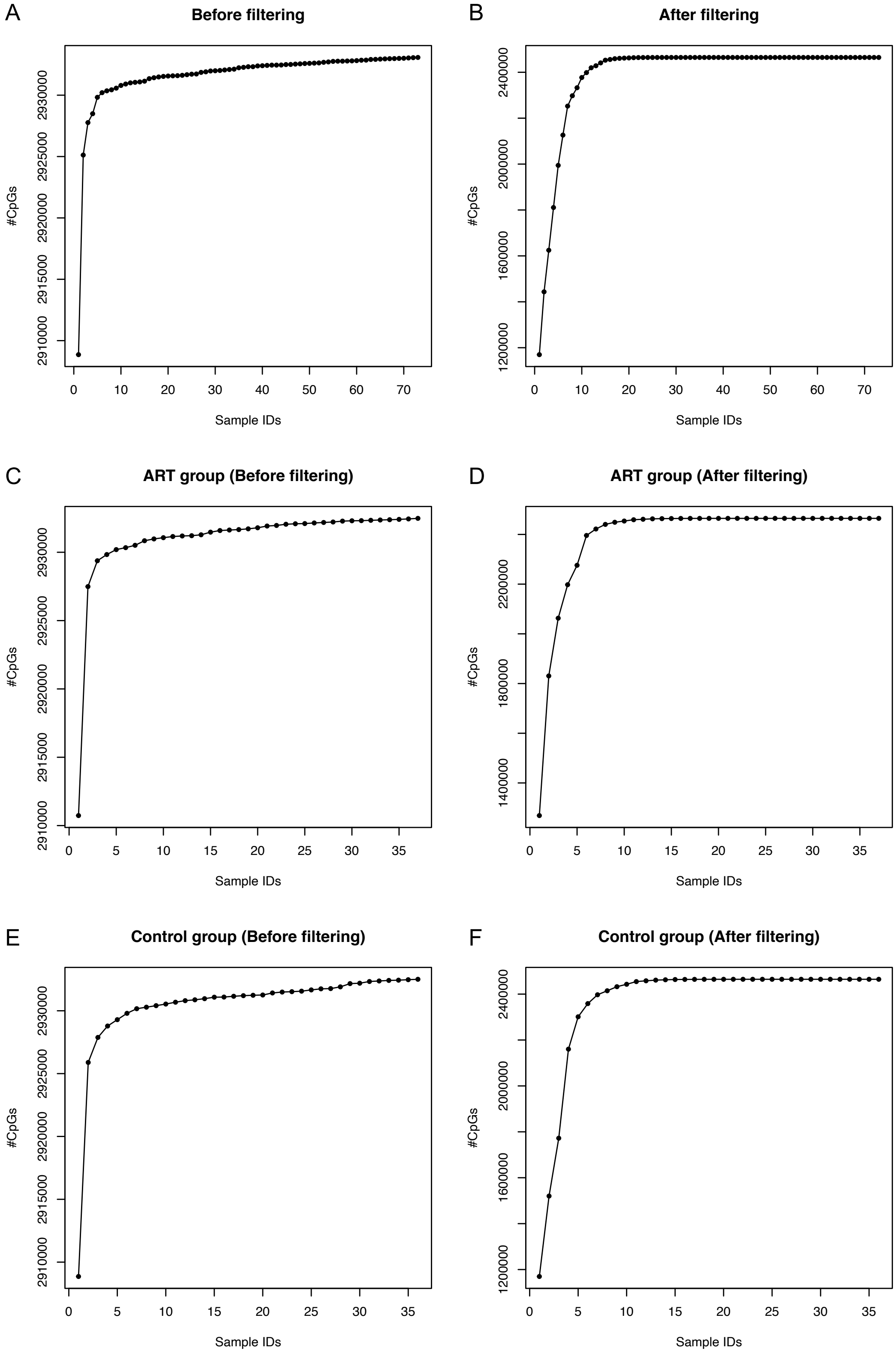

Figure S12: Cumulative distribution plot for CpG counts by sample size. (**A-B**). Cumulative distribution plot (CDP) for CpG counts by sample number before and after read coverage filtering (e.g. CpGs with read coverage <15X or >500X). (**C-D**) CDP for CpG counts by sample number before and after read coverage filtering for ART group samples. (**E-F**) CDP for CpG counts by sample number before and after read coverage filtering for control group samples. Samples were ordered by the total number of CpGs of the samples.

A

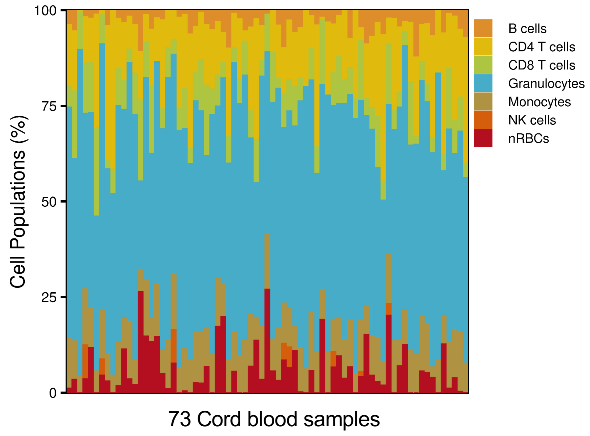

B

Cell type percent	Control<br>n = 36	ART/Hypofertile<br>n = 37	q-value
**Granulocyte (%)**	56.3 (9.0)	55.2 (11.5)	0.68
**CD8+ Tcell (%)**	6.7 (4.3)	9.7 (5.4)	0.08
**CD4+ Tcell (%)**	17.8 (9.2)	14.4 (8.9)	0.43
**Natural killer cell (%)**	0.4 (1.6)	0.7 (1.9)	0.68
**Bcell (%)**	3.6 (3.1)	3.2 (2.4)	0.68
**Monocyte (%)**	9.8 (3.3)	10.6 (3.0)	0.68
**Nucleated red blood cell (%)**	5.5 (5.8)	6.2 (7.2)	0.68

Mean (SD) shown and multiple unpaired t-tests performed to compare groups

Figure S13: Cell type composition in cord blood. (**A**) The cell type proportions for granulocytes, CD8+ T-cells, CD4+ T-cells, natural killer (NK) cells, B cells, monocytes and nucleated red blood cells (nRBC) were estimated and scaled for cord blood samples. For details regarding the deconvolution method, refer to Methods section. (**B**) Cell type composition was compared between control and ART/hypofertile group, with no significant differences identified.

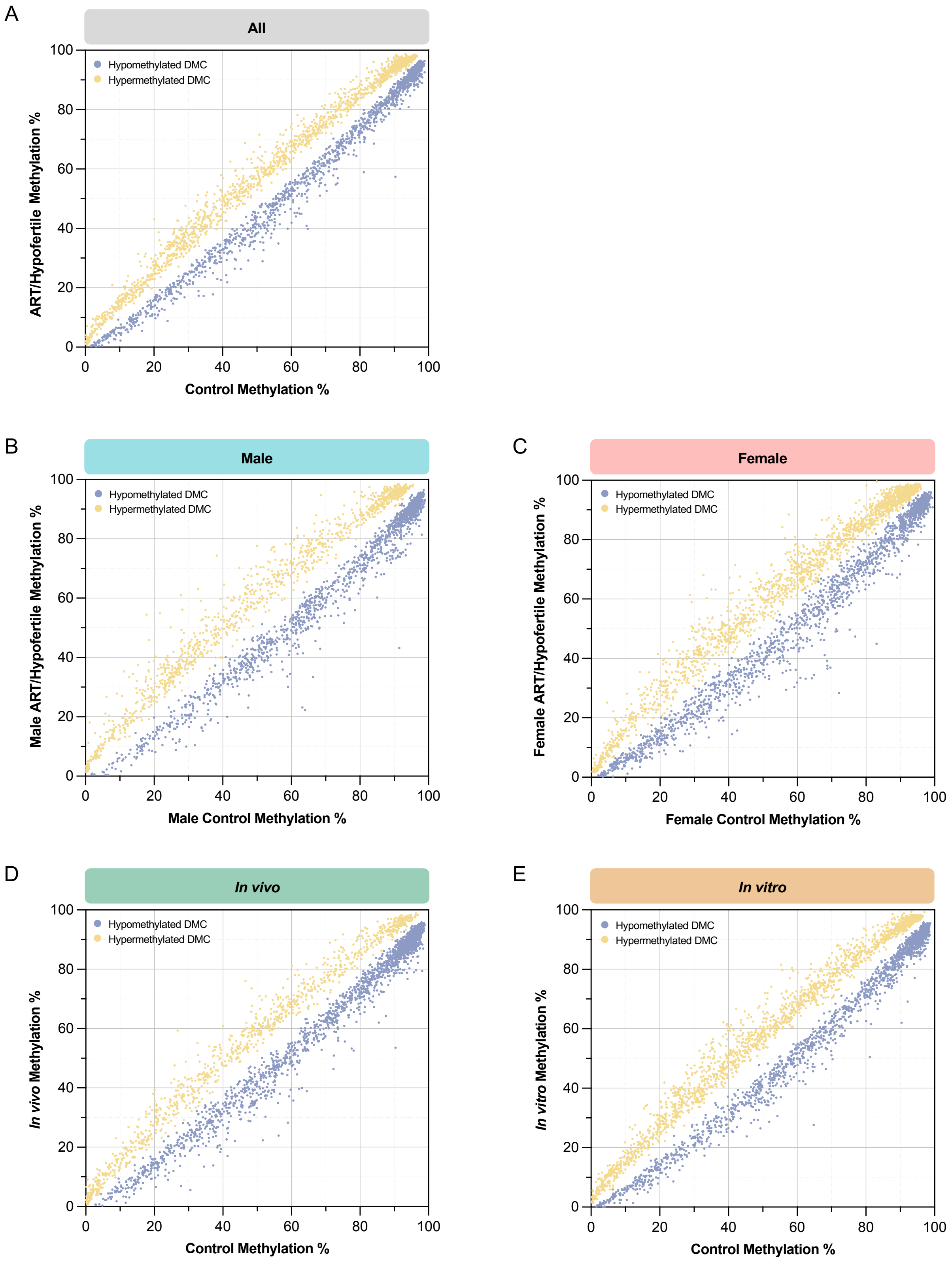

Figure S14: Methylation values of DMCs for all comparisons. DNA methylation values at the DMCs for all groups are shown for the following comparisons: (**A**) ART/hypofertile vs control (combined analysis), (**B**) ART/hypofertile vs control (male infants only), (**C**) ART/hypofertile vs control (female infants only), (**D**) *In vivo* vs control and (**E**) *In vitro* vs control. Each dot represents a differentially methylated CpG (DMC).
